# Chemical Editing Reveals Atomic-Level Control of Supramolecular Structure in Self-Assembling Peptides

**DOI:** 10.1021/jacs.6c11510

**Published:** 2026-09-12

**Authors:** Abhinaba Das, Ayisha Zia, Gunnar N. Eastep, Fengbin Wang, Vincent P. Conticello

**Affiliations:** † Department of Chemistry, 1371 Emory University, Atlanta, Georgia 30322, United States; ‡ Biochemistry and Molecular Genetics Department, 9967 University of Alabama at Birmingham, Birmingham, Alabama 35233, United States; § The Robert P. Apkarian Integrated Electron Microscopy Core (IEMC), Emory University, Atlanta, Georgia 30322, United States

## Abstract

Can replacement of single atoms within amino acid sequences redirect peptide self-assembly into different supramolecular structures? To address this question, we investigated a compositionally similar class of bola-amphiphilic peptides that were designed to self-assemble into filamentous nanostructures. The chemical differences between these peptides were confined to minimally perturbative substitutions on a phenylalanine side chain at a single site within the sequence. Cryo-EM structural analysis of five peptide filaments at near-atomic resolution revealed the presence of distinct supramolecular architectures between the different peptides. Despite sharing a common cross-β framework, the filament structures displayed distinct helical symmetries, protofilament organizations, and steric zipper interfaces. We hypothesize that the observed structural divergence between filaments arises from subtle changes in side-chain polarity and solvent interactions that remodel peptide packing at structural interfaces within cross-sectional amyloid layers. These findings demonstrate that the supramolecular structural landscape of self-assembling peptides is sensitive to atomic-level substitution and suggest that chemical editing provides a strategy for interrogating and potentially controlling biomolecular assembly. These results further highlight a fundamental limitation in the predictive design of peptide-based materials in that subtle chemical modifications in local composition can produce disproportionate changes in higher-order structure.

## Graphical abstract

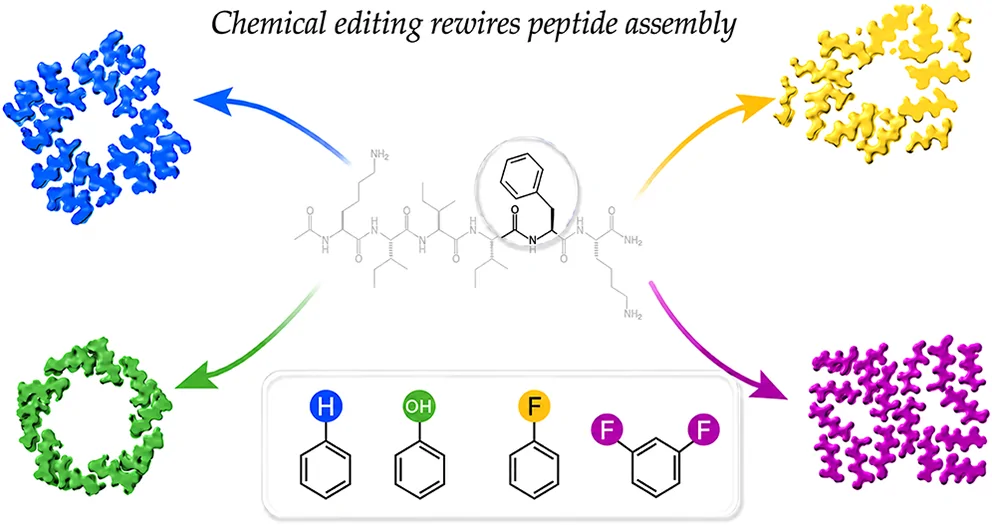

## Introduction

Peptide-based assemblies have long been investigated as synthetic platforms for the development of functional biomaterials.^1–3^ The technological appeal of these materials lies in the promise of encoding supramolecular structure within sequence information, potentially enabling the fabrication of functional assemblies with nanoscale precision. Predictive control over supramolecular structure would greatly facilitate the development of peptide-based materials^4^ for applications such as tissue engineering,^5^ controlled release,^6^ theranostics,^7^ vaccine development,^8^ and catalysis.^9^ These applications often depend on specific functional attributes, such as controlled degradation rates, selective cellular adhesion, tailorable mechanical response, and molecular recognition. Such properties are ultimately governed by supramolecular structure, in particular, interfacial interactions between subunits within the biomaterial as well as with heterologous substrates. However, despite substantial progress, the rational and predictive design of supramolecular architectures in peptide-based materials remains a major challenge.^10^ Like protein folding, the design of synthetic self-assembling peptides has been predicated on the hypothesis that information encoded within amino acid sequences can reliably direct hierarchical self-assembly into structurally defined architectures that enable functional control. Because synthetic self-assembling peptides generally exhibit less complex macromolecular architectures than native proteins, it was anticipated that these design principles could be used to create peptide sequences that self-assemble into user-defined supramolecular structures.

High-resolution structural analyses of synthetic peptide assemblies have exposed important limitations in current design paradigms.^11,12^ These studies demonstrated that intermolecular interactions programmed into designed peptide sequences often gave rise to emergent structural interfaces.^13–15^ The inability to accurately predict supramolecular structure has profound implications for the intentional design of functional materials. For example, KFE8,^16,17^ an amphipathic oligopeptide^18^ containing alternating hydrophobic and hydrophilic residues, was predicted to self-assemble into a supramolecular structure based on cross-β bilayer fibrils. Cryo-EM structural analysis of the resulting filaments confirmed the predicted formation of cross-β interactions between peptides. The latter interactions derived from richly designable structural interfaces corresponding to stable attractors in design space. These interfaces occurred frequently in statistical analyses of native protein interfaces in the Protein Data Bank (PDB)^19^ and corresponded to structurally preferred tertiary motifs (TERMs) that were effectively predicted from sequence information.^20^ However, cryo-EM analysis revealed a more complex structural landscape at larger length-scales in which additional inter-protomer interactions emerged that could not have been predicted *a priori*.^15^

These limitations highlight the need to develop a deep-seated knowledge of the relationship between amino acid sequence and supramolecular structure. One approach to this problem is to perform comparative structural analyses at high-resolution between peptide assemblies derived from closely related amino acid sequences.^13,14^ The structural information gained from these studies could be employed to iteratively improve design principles. Conceptually, this strategy is analogous to site-directed mutagenesis, which has long been used to probe the structural and functional roles of individual amino acids in native proteins. However, since most self-assembling peptides result from *de novo* design, meaningful structural comparisons are challenging to establish between sequence variants since a natural or “wild type” sequence would not be available to serve as a benchmark. Moreover, due to their compact size, conservative amino acid substitutions in designed self-assembling peptides would be expected to have amplified structural influence, potentially inhibiting meaningful comparisons between different peptide sequences.

We hypothesize that the introduction of single-atom or single-functional-group substitutions could provide a more fine-tuned method to interrogate the influence of local sequence changes on supramolecular structure. In native proteins, conservative atomic-level substitutions often preserve the overall fold while modulating folding kinetics, thermodynamic stability, and local intermolecular interactions.^21–23^ These structural modifications remain under-explored for native proteins, vis-à-vis traditional site-directed mutagenesis, due to synthetic challenges related to position-specific replacement of canonical amino acids with near-cognate isosteres.^24,25^ In contrast, solid-phase synthesis can straightforwardly introduce chemically similar amino acid analogues with site specificity into artificial peptide sequences. We hypothesize that atomic-level substitutions within designed oligopeptides could potentially induce significant changes in the kinetics of self-assembly and the resultant supramolecular structures, especially if interfacial interactions between subunits are physically altered in the resultant assemblies.^26–30^

To test this hypothesis, we investigated the influence of chemical editing on supramolecular structure within a class of self-assembling bola-amphiphilic peptides,^31^ Ac-Lys-Ile-Ile-Ile-Xaa-Lys-NH_2_ (Scheme 1). These surfactant-like peptides represent attractive building blocks for biomaterials development^32^ due to a strong propensity of the central domain to drive self-assembly into filamentous nanostructures through the hydrophobic effect. While bola-amphiphilic peptides tolerated substitutions within the hydrophobic central block, the supramolecular structure displayed sensitivity to conservative sequence changes.^33,34^ For example, Ile → Leu substitution at Xaa resulted in a significant morphological shift from wide-diameter tubes to narrow-diameter filaments.^34,35^

**Scheme 1:**
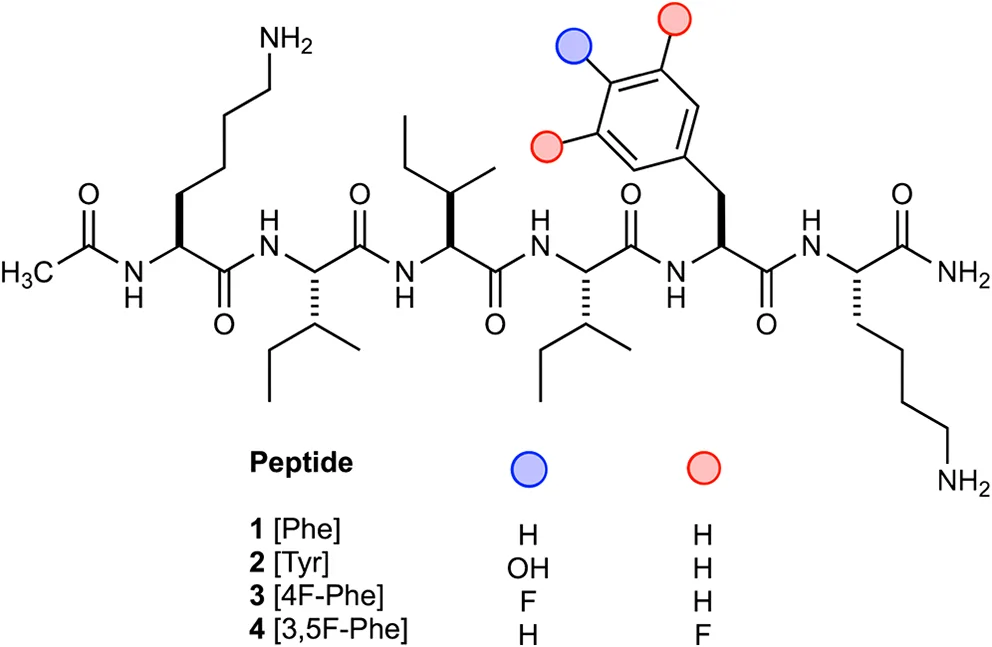
Chemical Structure and Sequences of Bola-Amphiphilic Peptides **1-4** That Were Employed in This Study

To more finely assess the potential structural impact of amino acid substitutions, four bola-amphiphilic peptide sequences (Scheme 1) were prepared in which chemically similar aromatic amino acids were introduced at the structurally sensitive Xaa site. We defined conditions in which each peptide self-assembled into high aspect-ratio filaments. Cryo-EM structural analyses were performed at near-atomic resolution^11^ on five peptide filaments derived from these four sequences. The resulting atomic models provided compelling evidence that minimally perturbative chemical substitutions in peptide sequence significantly altered supramolecular structure despite superficial similarity in filament morphology. Moreover, we demonstrate that the structures of the resultant peptide assemblies were based on amyloid-like cross-β fibrils.^36^ As previously noted for biologically derived amyloid structures, the steric-zipper packing interfaces within these synthetic peptide-based assemblies were sensitive to minor alterations in amino acid sequence, which may account for the observed difference in supramolecular structure between the filaments.

## Results and Discussion

The presence of structurally distinct residues at the fifth (Xaa) position of bola-amphiphilic peptide sequences Ac-Lys-Ile-Ile-Ile-Xaa-Lys-NH_2_ had been previously demonstrated to promote formation of narrow-diameter peptide filaments.^33,34^ The latter assemblies represent the preferred substrates for cryo-EM helical reconstruction.^11,37^ Recently we reported the cryo-EM structural analysis at near-atomic resolution (2.7 Å) of a peptide filament (PDB: 9CZ3) derived from self-assembly of the bola-amphiphilic sequence Ac-Lys-Ile-Ile-Ile-Leu-Lys-NH_2_ (**L5**, Ac = acetyl).^35^ While the **L5** peptide displayed controllable self-assembly behavior and a well-defined supramolecular structure, its sequence was not easily amenable to atomic-level substitutions at the Xaa position due to the limited availability of appropriately modified leucine analogues.

Instead, we opted to investigate sequence variants of a previously reported bola-amphiphilic peptide **1**, Ac-Lys-Ile-Ile-Ile-Phe-Lys-NH_2_ (Scheme 1),^33^ in which the near-isostere phenylalanine replaced the leucine residue in **L5**. The rationale for the selection of peptide **1** as the initial substrate for this study was based on the following considerations. First, the sequence of **1** conserved the design principles that promoted self-assembly of the parent **L5** peptide.^33^ Previous reports indicated that peptide **1** self-assembled into thin filaments of similar diameter (∼6 nm) to those observed for **L5** under the same self-assembly conditions. Second, the central hydrophobic block of peptide **1** encompassed β-branched Ile and aromatic Phe residues, which would be expected to promote β-sheet formation, as observed for **L5**.^38^ The amphiphilic triblock architecture of **1** was predicted to undergo nano-scale phase segregation of the terminal charged residues from the central hydrophobic core, which would result in sequestration into spatially distinct domains within the supramolecular structure. In addition, biophysical analyses of amyloidogenic peptide sequences, e.g., Ac-KLVFFAE-NH_2_ (Aβ ^16–22^ ),^39^ suggested that the presence of residues with aromatic side chains should stabilize the cross-β fibril structure through the potential formation of π–π interactions at internal packing interfaces between protomers.^40–42^ Aromatic stacking interactions have been frequently observed within amyloid structures. Finally, in contrast to **L5**, the Phe residue of peptide **1** is easily amenable to atomic substitutions on the aromatic nucleus through synthetic incorporation of commercially available phenylalanine analogues.

To interrogate the influence of atomic-level substitutions on supramolecular structure of amyloid-like peptide filaments, a small library of sequences derived from peptide **1** was chosen for investigation (Scheme 1). Peptide **1** served as the benchmark sequence due to its previously reported ability to self-assemble into high aspect-ratio, narrow-diameter filaments.^33^ Three structurally related peptide sequences, **2**, **3**, and **4**, were designed in which atomic substitutions were introduced onto the aromatic ring of phenylalanine. In each sequence variant, the atomic substitutions on the aromatic residue were located at positions on the side chain that were directed away from the peptide backbone (Scheme 1). Therefore, it was expected that these structural modifications would minimally perturb the predicted β-sheet conformation, which we hypothesized was structurally critical for peptide self-assembly.

Peptide **2** resulted from substitution of a Tyr residue for the original Phe residue of **1**, that is, of a proteinogenic amino acid for a structurally related one. Pairwise substitutions between Tyr and Phe have been thoroughly investigated for native proteins,^43^ in which the relative influence of this substitution on protein stability depends on its surrounding structural context within the native fold. While the influence of Phe versus Tyr substitutions has been examined for a limited number of self-assembling peptide systems,^27–29,44–48^ direct structural comparisons between otherwise compositionally identical peptides have not been reported at near-atomic resolution. Both Phe and Tyr occur frequently in the aggregation-prone regions (APRs) of amyloidogenic protein sequences.^49–51^ Moreover, peptide **2** had been previously reported to self-assemble into thin filaments of similar morphology to those derived from **1**.^33^ However, comparative structural analyses of the corresponding peptide filaments could not distinguish significant structural differences between the two filaments due to the limited resolution of the analysis.^33^

In peptides **3** and **4**, fluorine atoms replaced hydrogen atoms at different positions on the aromatic ring of the Phe derivative. The 4-fluorophenylalanine (4F-Phe) replacement in peptide **3** enabled a direct comparison of the structural influence of isologous substitutions at the *para* position on the aromatic ring. The C–F bond of 4F-Phe retained the polar character of the C–OH bond of Tyr but should display much weaker activity as a hydrogen bond acceptor.^52^ Peptide **4** incorporated 3,5-difluorophenylalanine (3,5F-Phe) into the sequence in place of Phe, thereby providing an opportunity to assess the influence of the number and placement of the fluorine substituents on supramolecular structure. Fluorinated phenylalanine derivatives have been previously demonstrated to influence the kinetics of peptide assembly and the morphology of the resultant filaments.^29,30,53,54^ However, as for the corresponding Phe→Tyr substitutions, the structural influence of Phe → 4F-Phe substitutions within the corresponding peptides sequences could not be evaluated due to unavailability of structural information at near-atomic resolution.

Peptides **1-4** were prepared using solid-phase peptide synthesis (Figures S1–S4) and assembled in aqueous solution (32 mM) using preparative conditions previously described for the parent peptides.^33,34^ Negative-stain transmission electron microscopy (nsTEM) was employed to monitor the reaction mixtures for the presence of self-assembled species at ambient temperature. In all cases, the presence of filamentous nanostructures was observed over an incubation period of 1 week (Figure S5). Peptides **1-3** formed narrow diameter filaments, while peptide **4** formed wide diameter nanotubes that displayed pronounced polymorphism in diameter. Thermal annealing of these nanotubes resulted in conversion to a population of self-assembled species that was enriched in narrow-diameter twisted filaments. The latter species superficially resembled the filaments derived from the assembly of peptides **1-3** at ambient temperature (Figure S5). Circular dichroism spectropolarimetry of the peptide solutions confirmed that filament formation coincided with the development of β-sheet conformation (Figure S6).

To understand the structural differences between the different peptide assemblies, filament preparations from **1-4** were subjected to cryo-EM structural analysis (Figure 1A-D). Representative cryo-EM images of the respective peptide filaments were consistent with the nsTEM imaging data. Reference-free 2D classification of the complete set of extracted segments was employed to quantify the population of each self-assembled species based on particle counts from the images (Figures S7–S8). These analyses indicated that the most populated 2D classes for each peptide sample corresponded to thin filaments that served as the basis for 3D reconstruction. After high-resolution cryo-EM data collection, standard helical refinement procedures were employed to determine the structures of five peptide filaments, including two distinct polymorphs derived from peptide **2**. Helical symmetry was determined using the trial-and-error method of assigning Bessel orders to two independent layer lines in the averaged power spectra.^11^ In each case, the correct solution yielded an EM density map in which the peptide densities were clearly separated and displayed the characteristic zigzag backbone conformation of β-strands in which the protruding side chain densities were consistent with the respective amino acid sequences (Figure 1E–N). The 3D reconstructions of the corresponding peptide filaments were determined at the following global resolutions (Figures S9–S13, GS-FSC 0.143 criterion): 3.50 Å (**1**), 3.66 Å (**2**, C5), 3.70 Å (**2**, C4), 3.3 Å (**3**), and 3.8 Å (**4**) (Table S1).

**Figure 1:**
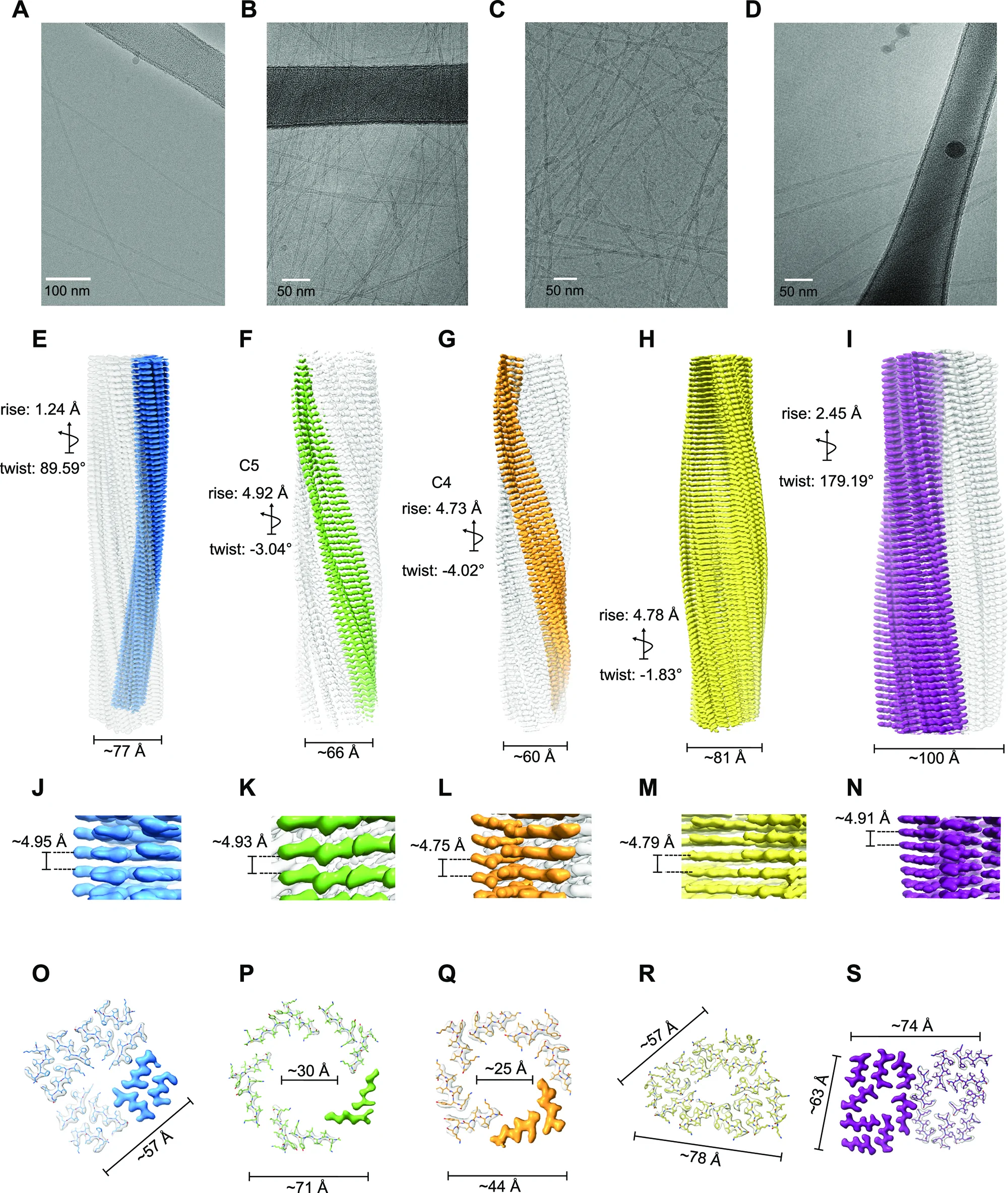
Cryo-EM structural analyses of bola-amphiphilic peptides (Ac-Lys-Ile-Ile-Ile-Xaa-Lys-NH_2_) reveal the formation of a structurally diverse family of filaments. (A–D), Representative cryo-EM micrographs: (A), peptide **1** (Xaa = Phe); (B), peptide **2** (Xaa = Tyr); (C), peptide **3** (Xaa = 4F-Phe); and (D), peptide **4** (Xaa = 3,5F-Phe). Scale bars are indicated (100 nm for A, 50 nm for B-D). The dark regions at the panel edges of the micrographs correspond to the carbon support film and were not included in particle extraction. (E–I**)**, Cryo-EM density maps from 3D reconstructions of the filaments derived from the respective peptides: E, **1**; F, **2**-C5; G, **2**-C4; H, **3**; and I, **4**. The helical parameters (rise and twist) and rotational point group symmetries are annotated for each reconstruction. Symmetry-related protofilaments are shown in grey with one protofilament highlighted for all structures except for peptide **3**, in which the ASU corresponds to the entire cross-sectional layer (H). (J–N), Close-up lateral views of the reconstructed densities revealing the characteristic cross-β fibril separation. The inter-strand distances are measured and annotated (ranging ∼4.7–4.9 Å). (O–S), Cross-sectional views of the atomic models fitted into the respective EM density maps. Key structural dimensions, including the lumen width and cross-sectional outer diameter, are annotated in Å.

Atomic models built into the density maps for the respective peptide filaments revealed the presence of cross-β fibrils as the main structural feature within each of the assemblies (Figure 1O–S). The axial spacings between peptides for all five filaments closely corresponded to the typical interstrand distance (∼4.8 Å) observed for hydrogen-bonded peptides within amyloid fibrils.^55–57^ In each case, the β-strands in the respective cross-β fibril were arranged in a parallel orientation as commonly observed in the structures of amyloid fibrils (Figure S14). Antiparallel models could not be accommodated satisfactorily in the respective cryo-EM maps. A comparison of the refinement statistics for a parallel versus an antiparallel model for the peptide **1** filament strongly supported parallel packing of β-strands (Figure S15).

The asymmetric units (ASUs) of the peptide assemblies defined protofilaments that coincided with helical symmetry elements, e.g., the presence of 4 protofilaments in the structure of the peptide **1** filament that propagated along the set of 4-start helices (Figure 1E). Collectively, the structures of these filaments resembled that of the previously reported **L5** filament in which peptides within cross-sectional layers packed tightly through the formation of amyloid-like steric zipper interfaces (Figure 2A–E). Cohesion between stacked ladders of the cross-sectional layers was mediated through a combination of hydrogen-bonding interactions and hydrophobic stacking within cross-β fibrils. Surprisingly, all four possible topological classes of parallel, antifacial β-sheets^58^ were observed across the set of steric zipper interfaces in the filaments, including the relatively uncommon class 3 topology (Figure 2A–E).^59^

**Figure 2:**
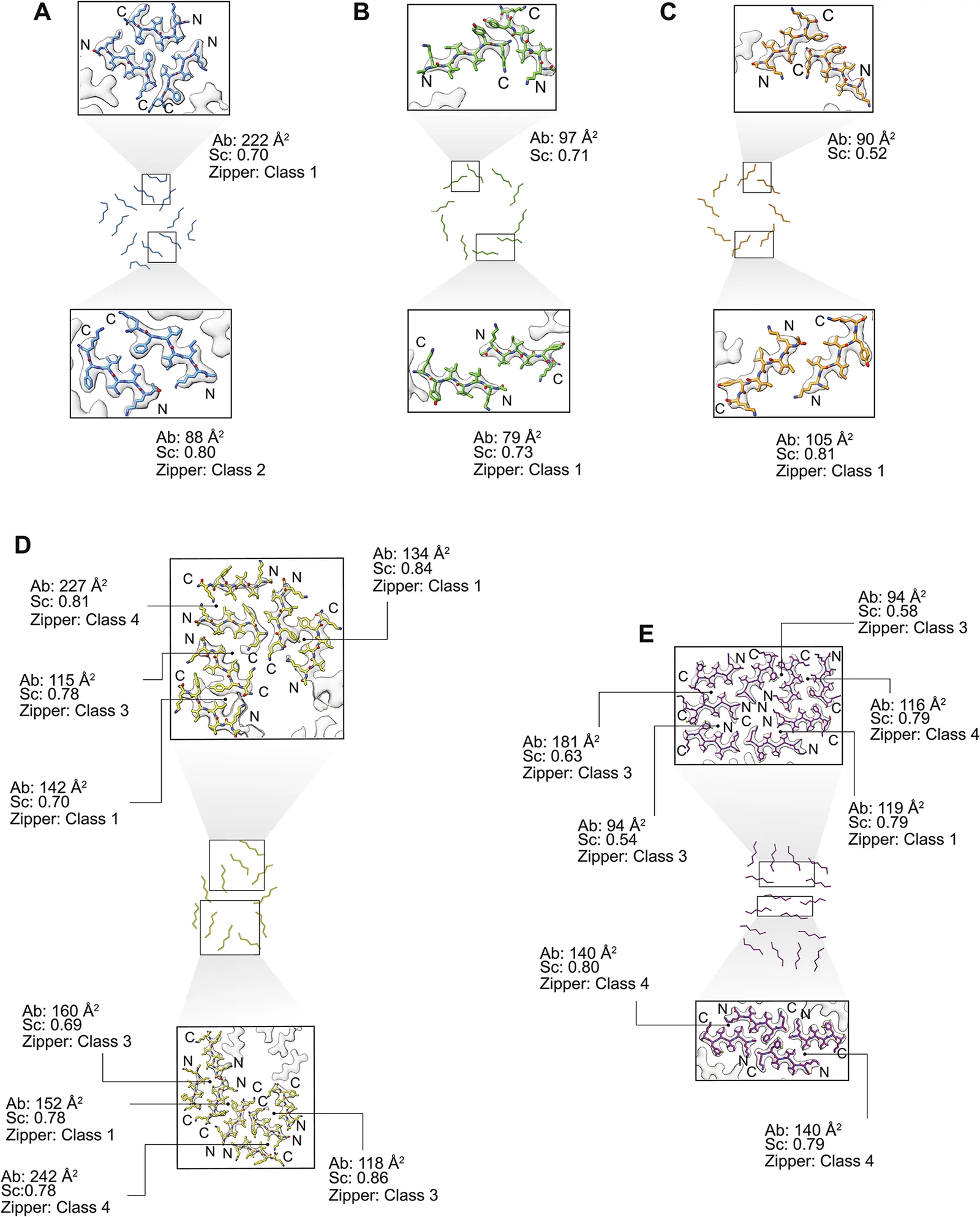
Structural analyses of interfacial interactions within cross-sectional layers of bola-amphiphilic filaments indicate the formation of amyloid-like steric zipper interfaces for **1** (A), **2**-C5 (B), **2**-C4 (C), **3** (D), and **4** (E). The central diagram depicts a cross-sectional layer of the atomic model as a ribbon diagram. Callout boxes above and below the central diagrams depict the full atomic models (stick format) built into the EM density map for each structurally unique interface within a cross-sectional layer of the respective filaments. For each interface, the buried surface area (Ab), the shape complementarity score (Sc), and the steric zipper classification are identified.

Notably, the filaments derived from the different bola-amphiphilic peptide sequences displayed dramatic structural differences despite the common cross-β scaffold for individual fibrils within the protofilaments. In contrast to most structures of amyloid fibrils,^36^ the bola-amphiphilic filaments contained multiple peptides within the asymmetric unit. For each filament, positional polymorphism was observed in the conformation of peptides within the respective ASUs, including 12 distinct symmetry-independent conformations in the ASU of the peptide **3** filament (Figures 1R and 2D). In addition to positional polymorphism, packing polymorphism was observed between filaments derived from compositionally similar peptide sequences. The filaments differed in helical symmetry, the size of the asymmetric unit, and the packing of amino acid side chains at the steric zipper interfaces. The two most closely related structures corresponded to the C_4_ and C_5_ symmetric filaments of peptide **2**. In either case, the ASU was derived from a pair of peptides that interacted through a class 1 steric zipper interface. The structural polymorphism observed between these two filaments could be interpreted in terms of an alternative packing arrangement of four versus five structurally similar protofilaments that were related through C^5^ and C^4^ rotational point symmetry, respectively (Figure 1P,Q). The difference in the number of protofilaments between the C_4_ and C_5_ assemblies of **2** was manifested in a slight increase in rise (4.73 Å versus 4.92 Å) and a decrease in left-handed twist (−4.02° versus −3.04°), respectively, which presumably resulted from slight differences in side chain packing at the steric zipper interface to accommodate different numbers of protofilaments (Figure 2B–C).

Two distinct types of peptide–peptide interfaces were observed between the cross-β fibrils within the ASUs of the peptide filaments. The most prominent interactions corresponded to the formation of steric zipper interfaces between mated pairs of β-sheets in a face-to-face orientation (Figure 2A–E). For filaments derived from peptides **1**, **3**, and **4**, the steric zipper interfaces usually displayed shape complementarity (Sc) and buried area (Ab) values that were consistent with tightly mated sheet surfaces (Sc ≥ 0.7, Ab ≥ 120 Å^2^). The two polymorphs of peptide **2** represented exceptions to this general observation in that lower Ab values were observed for the Class 1 steric zipper interfaces although with relatively high Sc values. We attributed this difference to the backbone offset between the peptides at the steric zipper interface, which may have resulted from the need to exclude the polar hydroxyl group of the Tyr residue from the hydrophobic core (*vide infra*).

A second, related type of peptide–peptide interface was also observed in the ASU of every peptide filament, which involved face-to-edge interactions between two or three β-strands. The most common of these interfaces involved a π-shaped arrangement of three chains in which two β-sheets interacted through a face-to-face steric zipper interface, while a third chain was oriented at an angle, often nearly perpendicular to the other two, through a face-to-edge interaction. This π-like ternary interface was also observed in the ASU of the L5 filament structure.^35^ The binary face-to-edge interaction observed in the two structural polymorphs of peptide **2** (Figure 2B–C) may be a π-shaped interface in which a third strand was potentially prohibited from association. Formation of this face-to-face interaction, similar to that observed in the ASU of the peptide **1** filament (Figure 2A), would have necessitated burial of polar hydroxyl groups from the Tyr side chain of **2** in a low dielectric environment at an inter-strand interface.

We hypothesize that these ternary interfaces corresponded to stable structural nodes that promoted further interactions between peptides located within and between ASUs. As such, they represented critical features that contributed to the structural integrity within cross-sectional layers of the filaments. The capping strands of the π-shaped interfaces were often involved in the steric zipper interactions with additional peptides, as in the peptide **1** filament, in which one such interface occurred between structurally adjacent ASUs (Figure 2A). Formation of these π-shaped interfaces might have arisen from structural features implicit within the design of these bola-amphiphilic peptide sequences (Scheme 1). The block-like architecture of these peptides places the hydrophobic residues at positions 2 and 5 at solvent-exposed edges of the bilayer β-sheets after formation of a steric zipper interface. This surface pre-organization provided an opportunity for nucleation of an additional interfacial interaction between the edge of the bilayer and the face of a third cross-β fibril. We posit that the formation of these interactions would be strongly favored for these peptide sequences based on their structural conservation within this class of peptide filaments. Indeed, within the peptide **1** filament (Figure 2A), the outer β-strand of the bilayer β-sheet distorted its backbone conformation to accommodate formation of a ternary interface in the ASU, which argued strongly for the favorability of these π-shaped interfaces. This chain kinking, which was reminiscent of that observed in cross-β fibrils from low-complexity aromatic-rich kinked segment (LARKS) motifs,^60^ resulted in the formation of a hydrophobic cluster derived from residues from the three peptide chains in the ASU. Similar π-shaped face-to-edge interactions were often observed at internal structural interfaces within the β-amyloids in the Amyloid Atlas.^36,61^ High-resolution structural analyses of amyloid fibrils indicated that longer polypeptide chains typically folded into a serpentine conformation within cross-β layers, in which aggregation prone regions (APRs) interacted through steric zipper packing interfaces. This convoluted packing arrangement often involved the formation of turns that were stabilized through face-to-edge interactions like those observed within the bola-amphiphilic peptide filaments (Figure 2 A–E).

The presence of similar interstrand interfaces between compositionally related but structurally distinct peptide-based filaments raised several intriguing questions. The first concerned the relationship between the π-shaped structural interfaces, local peptide conformation, and the chemical identity of the phenylalanine derivative. To address this question, the conformations of symmetry independent peptides within the ASUs of the bola-amphiphilic peptide filaments, including the previously reported **L5** structure (PDB: 9CZ3),^35^ were mapped onto a pseudo-phylogenetic tree based on differences in backbone RMSD for the Cα alignments in ChimeraX (Figure 3A).^62^ While conformational clustering into structurally related clades was observed, the different peptide sequences were diversely distributed across the pseudo-phylogenetic tree with conformational mixing occurred across the six filament structures. Moreover, the π-shaped interfaces did not appear to display a conformational preference based on the absence of cluster formation in the tree. In addition, of the limited number of steric zippers involved in the π-shaped interfaces, a preference was observed for class 4 with four of six instances, although examples of class 1 and class 3 were also observed. So, while the presence of full or partial π-shaped interfaces was a common structural feature among these bola-amphiphilic peptide filaments, its formation appeared compatible with a wide distribution of peptide conformations and a range of steric zipper interfaces.

**Figure 3:**
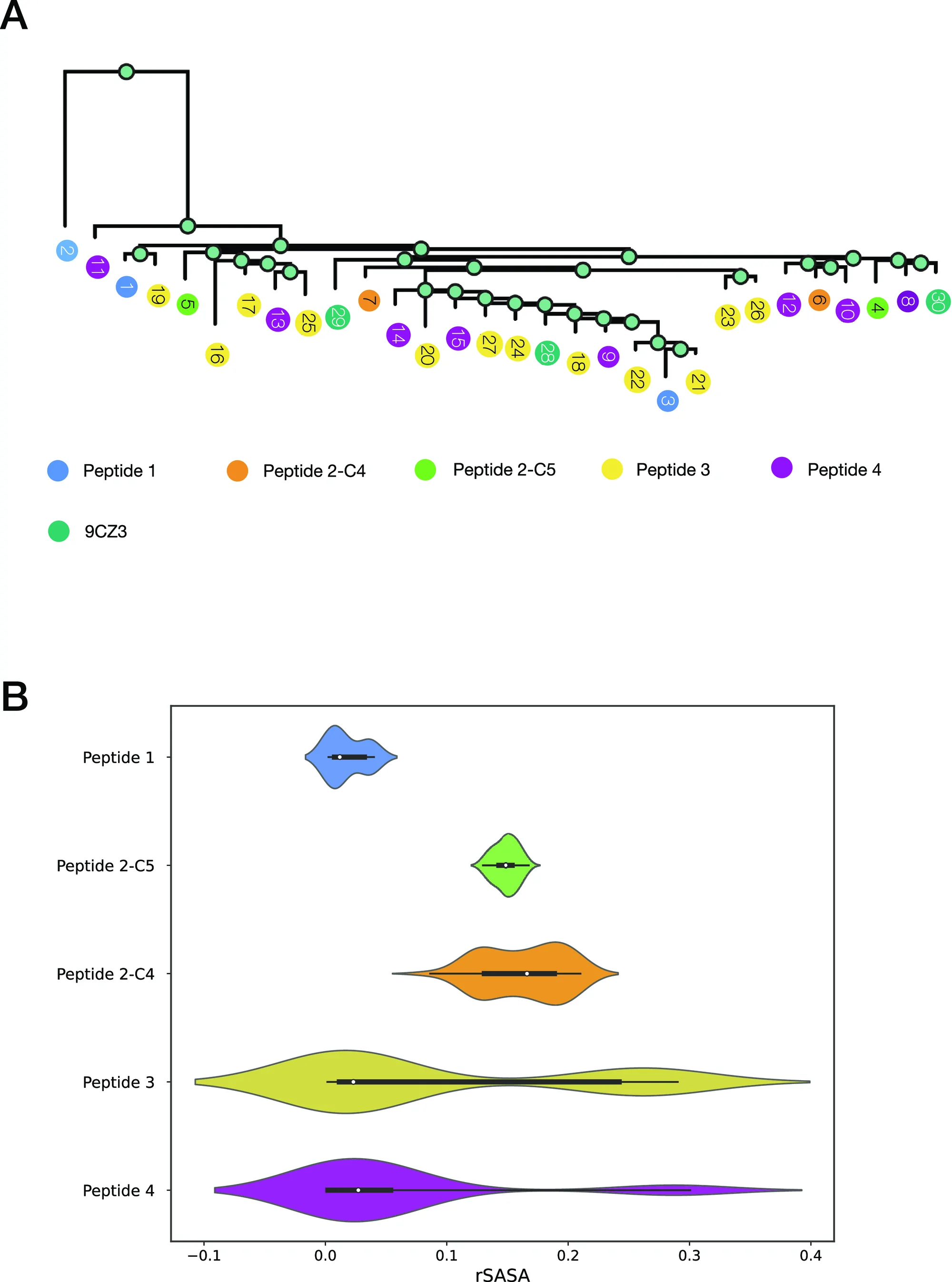
Structural comparisons of local conformation and side chain packing within the cross-sectional layers of the filaments. (A). Pseudo-phylogenetic tree illustrating the structural relationships between the backbone conformations of symmetry independent peptides within the ASUs of bola-amphiphilic peptide filaments. The tree was constructed based on Cα backbone root-mean-square deviation (RMSD) and includes the L5 structure (PDB ID: 9CZ3)^35^ for comparison. A full identification of the peptide conformation for each representative of the pseudo-phylogenetic tree is provided in Figure S16. (B). Violin plot comparison of the relative solvent accessible surface area of aromatic residues within cross-sectional layers of bola-amphiphilic peptide filaments.

A second question focused on whether the large differences in supramolecular structure between the filaments could be rationalized based on the influence of the chemical substitutions on peptide packing at the steric zipper interfaces. Notably, the aromatic side chains were arranged in a ladder-like array in each of the filaments. Aromatic ladders^63^ have been previously reported for amyloid structures in which the π-stacking interactions were proposed to contribute to the stability of the fibril but were not necessary for its formation.^49,50^ The centroid-to-centroid distances between aromatic rings in the respective ladders ranged from 4.7 Å to 5.3 Å, which were consistent with the axial rise of the cross-β fibril. Based on the observed distances between aromatic side chains, intra-sheet and inter-sheet π–π interactions appeared to contribute minimally to the stability of the respective filaments. The main difference between the aromatic residues in peptides **1-4** corresponded to substitutions that altered the polarity and H-bonding potential at side chain positions that were distal from the main chain. In a β-strand conformation, the aromatic substituents extend outward from the sheet face, potentially engaging with residues on the surface of other sheets or with aqueous solvent. To gain insight into the local environment of the different aromatic groups, the relative solvent accessible surface area (rSASA) was determined and plotted for each filament (Figure 3B). Distinct differences were observed in rSASA between aromatic residues within and between the different filaments.

The most pronounced chemical difference between the aromatic residues corresponded to the substitution of Tyr for Phe in peptide **2** versus peptide **1**. A comparison of rSASA values indicated that the Phe residues in the peptide **1** filament were completely buried within the hydrophobic core of a cross-sectional layer. In contrast, the Tyr residues were partially exposed at the solvent interface (Figure 3B). Presumably, the structural differences observed between the filaments derived from these two peptides, particularly the number of peptides in the ASU, arose from chemical differences between Phe and Tyr. The ASU of the Phe filament consists of 3 β-strands engaging in hydrophobic interactions at a π-shaped interface. The structures of the ASUs for the two Tyr filaments were based on two β-strands involved in a single face-to-edge interaction. The localization of Tyr at solvent exposed surface sites was presumably due to the presence of the hydroxyl substituent. The presence of this group presumably accounts for the large difference in Kyte-Doolittle hydrophobicity^64^ between Tyr (−0.27) and Phe (1.10). The formation of a filament **1**-like ASU would bury at least one polar tyrosine residue at an internal hydrophobic site. In contrast, the hydrophobic side chains of the Phe residues can be accommodated within a larger ASU containing a greater number of peptide chains.

While the Phe to Tyr substitution in peptide **2** significantly altered supramolecular structure of the corresponding filaments, it did not preclude self-assembly despite the greater polarity of the latter residues.^33^ This observation suggested that more structurally conservative chemical substitutions might promote filament formation and provide further insight into the relationship between peptide composition and supramolecular structure. Fluorine has often been employed as a chemical surrogate for hydrogen atoms in organic and biomolecules.^65,66^ The strong C–F bond and its minimally disruptive steric footprint implied that 4-fluorophenylalanine and 3,5-difluorophenylalanine could chemically bridge the gap in physico-chemical properties between phenylalanine and tyrosine in the bola-like peptide sequences without disrupting its conformational preference for β-sheet formation or its propensity for self-assembly into a filament.

Peptides **3** and **4** were expected to be more hydrophobic than peptide **1** since fluorine atom substitutions on aromatic rings display a small positive Hansch hydrophobicity value^67,68^ in comparison to hydrogen atoms. Based on this consideration, we expected that the hydrophobicity of the fluorine substitutions in peptides **3** and **4** might restore the structural phenotype observed in the peptide **1** filament. Surprisingly, the filament structures derived from both sequences indicated that a subset of fluorinated amino acids resided at surface-exposed sites with higher estimated rSASA values than for the corresponding tyrosine residues in the peptide **2** filaments. This effect was more pronounced for peptide **3**, in which 4 of the 12 symmetry independent peptides in the ASU of the filament had 4F-Phe residues located at surface-exposed sites. Hoffmann et al.,^69^ recently reported an intrinsic hydrophobicity scale for amino acids based on the quantity α, which is a measure of side chain packing efficiency in the gas phase. These measurements included mono-fluorinated phenylalanine derivatives in addition to the canonical amino acids. The observed trend in α value indicated that Phe (1.042) > 4F-Phe (1.014) > Tyr (0.998), which suggested that mono-fluorinated phenylalanine derivatives were less hydrophobic than Phe. The latter was tentatively attributed to the strong dipole moment of the aromatic C–F bond (μ­(C_6_H_5_F) ∼1.6 D),^52,70^ which potentially enabled it to engage in neutral dipole interactions with polar solvent molecules. Consequently, we hypothesize that fluorinated Phe derivatives may be able to more effectively partition between buried and solvent exposed sites than the corresponding Phe side chain.

In comparison, only one of the eight symmetry independent peptides in the ASU of the peptide **4** filament had a 3,5F-Phe residues located at a surface-exposed site. However, these filaments were formed only after thermal annealing at 95 °C, conditions under which the dielectric constant of water decreases significantly. In contrast, peptide **4** assembles into polymorphic, wide-diameter, thin-shell nanotubes at ambient temperature. The large morphological difference between ambient and annealed filaments from peptide **4** may arise from different solvent interactions between the 3,5F-Phe at ambient versus elevated temperature. A compositionally related bola peptide, Ac-Lys-Ile-Ile-Ile-Dopa-Lys-NH_2_ (DOPA: 3,4-dihydroxyphenylalanine), formed morphologically similar tubes at ambient temperature, presumably due to the presence of the more polar DOPA residue, providing further evidence that the presence of distal functional groups could alter supramolecular structure. In addition, Chowdary et al.,^30^ reported that substitution of 3,5F-Phe for Phe in the sequence of the amyloidogenic peptide hIAPP_22‑27_ (NFGAIL) resulted in an anomalously large increase in lag time for self-assembly in comparison to other fluorinated Phe derivatives, which was attributed to a potentially strong dipole interaction between 3,5F-Phe and solvent water molecules.

Finally, to quantify the energetic differences between filaments based on the different peptide sequences, we compared their standard free energies (Δ*G*°) of solvation per residue, per chain, and per cross-sectional layer (Figure 4). Since the fluorinated phenylalanine derivatives in peptides **3** and **4** were non-canonical amino acids, they could not be directly included in the calculations of solvation free energies. Instead, variant sequences were created in which phenylalanine was modeled in place of the non-canonical amino acids in the respective cross-sectional layers, which might have led to inaccuracies in the free energy calculations due to potential structural mismatches. Despite this caveat, several conclusions can be reliably drawn from this analysis. First, the solvation free energies per residue (Δ*G*°_residue_) for the filamentous structures were on the order of the corresponding per residue stabilities calculated from the structures of biologically derived amyloid assemblies (Figure 4B).^36,71^ In contrast to amyloid structures, the free energies of solvation per chain (Δ*G*°_chain_) were significantly less stabilizing, ranging from −3.05 to −6.03 kcal/mol (Figure 4C), than the corresponding values observed for typical amyloids (mean Δ*G*°_chain_ of ca. −24 kcal/mol).^72^ In a previous analysis of the **L5** peptide filament, we postulated that this discrepancy was due to the short chain length of the bola-like peptides vis-à-vis typical amyloid sequences. Despite this lower stability, all bola-amphiphilic peptides self-assembled into amyloid-like filaments in which typical steric zipper interfaces were observed that displayed high shape complementarity values. Notably, the Δ*G*°_layer_ values for all five filaments were within the median range of values observed for cross-sectional layers in amyloid fibrils. However, the bola-like peptide filaments achieve this stability from burial of multiple peptides, ranging from 8 to 16 subunits, within a cross-sectional layer (Figure 4CA rather than one or two long chains. The latter situation is more common for the long chains in the structures of biologically derived amyloids, independently of whether they were assembled *in vitro* or isolated *ex vivo*.

**Figure 4:**
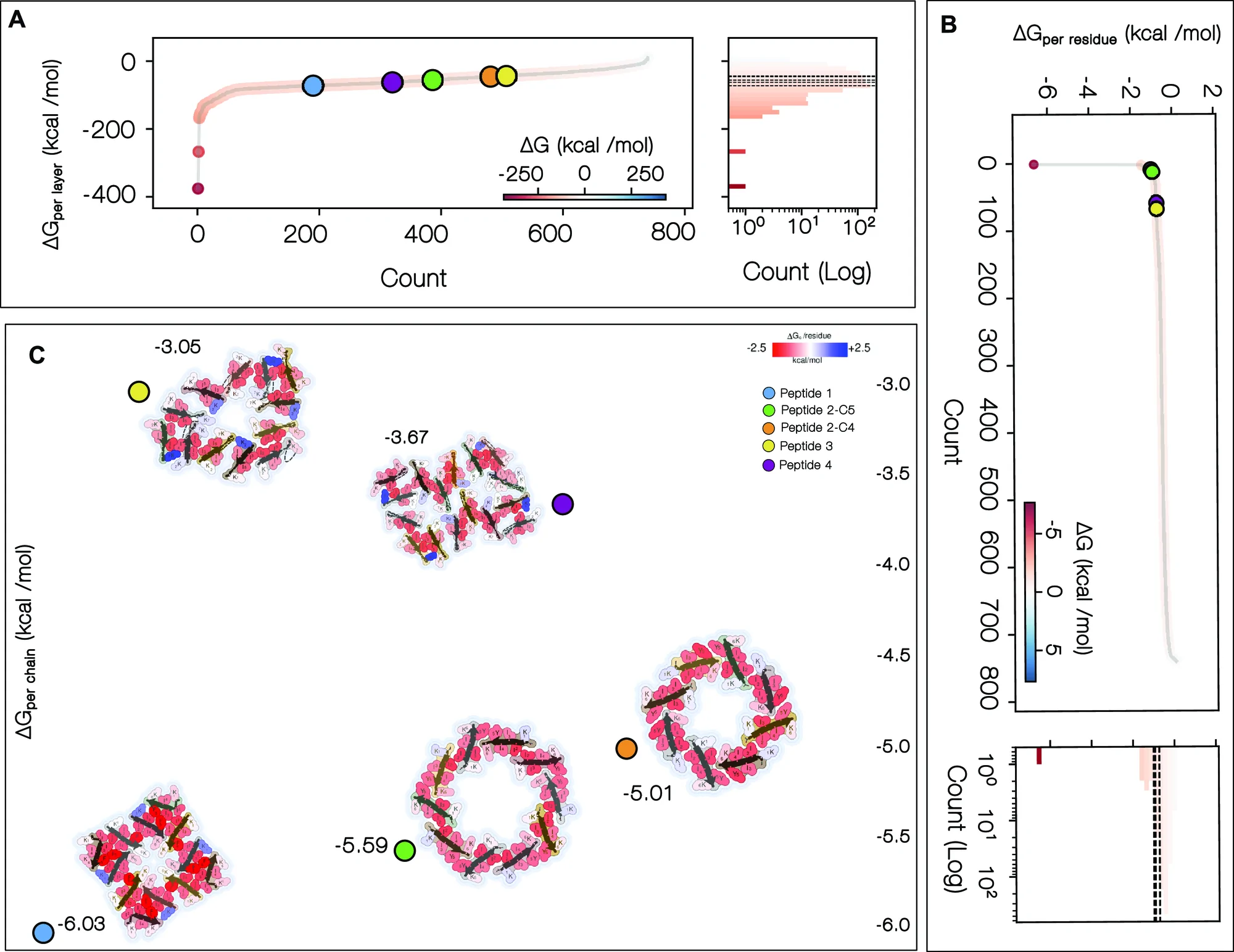
Comparison of the standard free energies (Δ*G*°) of solvation for filaments derived from peptide **1-4** with structurally characterized amyloids in the Amyloid Atlas.^61^ (A). Distribution of per-layer free energy Δ*G*°_layer_ across the amyloid structure dataset. The left panel displays the calculated energy per layer (kcal/mol) for a library of amyloid structures in the Amyloid Atlas. The data points are colored according to stability (red indicating favorable energy, blue indicating less favorable energy). Structures in the present study are highlighted in their corresponding colors in Figure 1 to illustrate their relative stability. The right panel shows a horizontal histogram of the energy distribution with frequency plotted on a logarithmic scale, highlighting the density of structures at various energy levels. The energy values of the solved structures are seen to cluster near the center of the distribution coinciding with the most populated stability region. (B). A similar analysis performed for ranked Δ*G*°_residue_ energy values. The left panel illustrates the ranked per-residue energy (kcal/mol) for the entire Amyloid Atlas dataset, highlighting the five structures in the current study. The right panel displays a frequency histogram of energy values on a logarithmic scale. The dashed lines indicate the energy levels of the five highlighted PDB entries showing their position within the wider population density. (C). Comparative solvation energy maps for reported cryo-EM assemblies. Structures are arranged vertically by their averaged Gibbs free energy per chain Δ*G*°_chain_ with the *x*-axis used solely for visual separation. Residues are colored by local stabilization energy, ranging from favorable (red, −2.5 kcal/mol) to unfavorable (blue, +2.5 kcal/mol). The plot reveals that assemblies with a π-shaped domain architecture display unfavorable values for Δ*G*°, which are localized at the termini of the mating β-sheets, due to partial burial of the charged lysine residues. The plots in Figure 4 were adapted with permission from reference 36. Copyright 2021 Elsevier.

## Conclusion

Our cryo-EM structural analyses provide strong evidence that semi-conservative chemical editing of self-assembling, bola-amphiphilic peptide sequences resulted in significant changes in supramolecular structure. Despite a common cross-β structural framework, filaments derived from these chemically similar peptides displayed distinct helical symmetries, protofilament organizations, and steric-zipper interfaces. Structural comparisons between filaments suggest that this sensitivity arises from subtle differences in side-chain polarity and solvent interactions that remodel peptide packing at critical inter-sheet interfaces within cross-sectional amyloid layers. Small changes in the chemical environment of aromatic residues alter the balance between buried and solvent-exposed interactions, ultimately redirecting the assembly pathway toward alternative supramolecular architectures.

More broadly, these findings highlight the current challenges inherent in the molecular design of peptide-based materials. While chemical editing provides a precise strategy for probing and potentially tuning peptide self-assembly, the pronounced structural consequences of seemingly minor substitutions underscore an important limitation in predictive nanomaterials design, namely, that local atomic-scale modifications cannot be assumed to preserve higher-order structure. Given that peptide-based materials are often modified to promote specific function, it is important to acknowledge that such substitutions may alter the structure and properties of the assemblies in ways that may be difficult to predict *a priori* using current design principles.

## Supplementary Material



## Data Availability

Refined structural models and cryo-EM density maps of the peptide filaments were deposited in the Protein Data Bank (PDB) and the Electron Microscopy Data Bank (EMDB), respectively, under the following accession codes: peptide **1** (PDB 9D2N, EMD-46501), peptide **2**-C5 protomer nanotube (PDB 9NFN, EMD-49371), peptide **2**-C4 (PDB 9NGK, EMD-49387), peptide **3** (PDB 13MF, EMDB-77153), and peptide **4** (PDB 13FM, EMDB-77045). All other data needed to evaluate the conclusions in the paper are present in the manuscript or the Supporting Information.

## Notes

Experimental materials and methods, HPLC, ESI-MS, CD spectra, nsTEM, FSC curves for cryo-EM analyses, and a table of cryo-EM data collection and refinement statistics (PDF)
