# Supplementary material for: Chemical Editing Reveals Atomic-Level Control of Supramolecular Structure in Self-Assembling Peptides
Source: J Am Chem Soc. 2026 Sep 12;148(37):40186–97. doi: 10.1021/jacs.6c11510 (PMC13614187; doi:10.1021/jacs.6c11510)
Supplement: Supplementary file 1 [file ja6c11510_si_001.pdf]

Supporting Information

Chemical Editing Reveals Atomic-Level Control of Supramolecular Structure in Self-Assembling Peptides

Abhinaba Das,[1] Ayisha Zia,[2] Gunnar N. Eastep[2], Fengbin Wang,[2] and Vincent P. Conticello[1,3]*

[1] Department of Chemistry, Emory University, Atlanta, GA, 30322, USA.

[2] Biochemistry and Molecular Genetics Department, University of Alabama at Birmingham, Birmingham, AL, 35233, USA

[3] The Robert P. Apkarian Integrated Electron Microscopy Core (IEMC), Emory University, Atlanta, GA, 30322, USA

*Correspondence:
vcontic@emory.edu (V.P.C.)

Contents

Experimental Methods……………………………………………………………………3-8

Supporting Figures…………………………………………………………………….9-27

Supporting Table……………………………………………………………………...28

Supporting References………………………………………………………………29-30

EXPERIMENTAL METHODS

Materials

Chemical reagents were purchased from Sigma-Aldrich Chemical Co. (St. Louis, MO) unless otherwise specified. Peptides were purchased from either GenScript USA, Inc. (Piscataway, NJ) or Synpeptide Co. Ltd. (Shanghai, China) as the N-acetyl, C-amide derivatives at ≥ 95% purity as assessed by analytical HPLC. All purchased chemical reagents and peptides were used without further purification.

Peptide assembly

Stock solutions were prepared by solubilizing the purified, lyophilized peptides in ddH_2O at a concentration of approximately 32 mM. After dissolution, the pH of the peptide solutions was adjusted to a value of ~4.0. The resulting solutions were incubated at ambient temperature for one week to achieve equilibrium. For all peptides, the initially clear solutions transformed into self-supporting hydrogels during incubation.

Circular dichroism (CD) spectropolarimetry

CD measurements were performed on a Jasco J-1500 CD spectropolarimeter using 0.20 mm thick quartz plates from Hellma USA Inc (Plainview, NY). Three spectra were collected and averaged in the wavelength range from 190 to 260 nm at a scanning range of 100 nm min^{-1} with a bandwidth of 2 nm and a data pitch of 0.2 nm. Stock solutions of peptides were diluted to a concentration of ~25 mM prior to the acquisition of CD data. Peptide concentration was determined spectrophotometrically at 214 nm using molar extinction coefficients calculated from the amino acid composition of the respective peptides.[1] CD spectra are reported as mean residue ellipticity (MRE) as a function of wavelength.

Negative-stain TEM analysis

TEM grids were prepared using 10-15-fold dilutions of peptides (32 mM) in aqueous solution at pH 4.0. Aliquots of the peptide solutions (4 µL) were deposited onto a 200-mesh carbon-coated copper grid from Electron Microscopy Services (Hatfield, PA). After 90 s of incubation on the grid, excess liquid was wicked away, leaving a thin film of sample. Uranyl acetate negative stain (4 µL of a 1% solution) was deposited onto the thin film. After 1 min, the remaining moisture was

wicked away, and the grid was dried overnight in a vacuum desiccator. Electron micrographs were captured on a JEOL JEM-1400 transmission electron microscope equipped with a LaB6 filament operating at an accelerating voltage of 80 kV.

Cryo-EM imaging and analysis

Filamentous assemblies of peptides **1**, **2**, and **4** were analyzed at the Robert P. Apkarian Integrated Electron Microscopy Core (RRID: SCR_023537) at Emory University. Aliquots (3-4 µL) of the peptide solutions were applied to a glow-discharged lacey carbon grid. The droplet was blotted for 4 s and plunge-frozen in liquid ethane using a Vitrobot™ Mark II plunge freezer. Grids were imaged on a Talos Arctica (200 keV, Thermo-Fisher Scientific) equipped with a K3 camera (Gatan). For the peptide **1**, a total of 1878 movies were collected under electron counting mode at 1.1 Å per pixel, using a defocus range of 1-2 µm with ~48 electrons per $Å^2$ distributed into 48 fractions. For peptide **2**, about 4000 movies were recorded in counting mode at pixel size of 0.84 Å per pixel with defocus range of 1-2.5 µm with ~57 electrons per $Å^2$ distributed over 48 fractions. Image processing and structural determination were performed using cryoSPARC[2] with the following workflow. Images were motion corrected using Patch Motion Correction and contrast transfer function (CTF) was estimated using Patch CTF estimation.[2, 3] Initial templates were generated by manual picking of filaments, followed by a round of 2D classification. Selected templates served as the input for Filament Tracer. The separation distance between particles was determined based on the expected axial rise to be used during reconstruction. Particle picks were selected after launching an Inspect Pick job. Extracted particles were downscaled, and non-peptide particles and particles in low-resolution class averages were separated by multiple rounds of reference-free 2D classifications. Particles having clear 2D averages indicative of underlying structural features were selected and re-extracted. Polymorphs were separated based on class average appearance and comparison of the averaged power spectra. For reconstruction of the 2D classes, all possible helical symmetries were calculated from the averaged power spectra of raw particles and were tested by trial-and-error assignment of Bessel orders to two independent layer lines. Helical symmetry was deemed correct if peptide-like features could be discerned within the reconstructed density map. These features included structural elements that were consistent with the presence of a β-sheet backbone conformation displaying clear separation between β-strands, and sidechain density consistent with the peptide sequence. The final volumes were then sharpened

using either local sharpening, DeepEMhancer[4] available in cryoSPARC or EMReady.[5] Data collection statistics are listed in Table S1.

For peptide **3**, the filament sample (4.5 µL, 20 µM) was applied to glow-discharged lacey carbon grids and plunge frozen using a Leica plunge freezer. Grids were screened under Glacios (200 keV, Thermo Fisher), and selected grids with thin ice were imaged on a Titan Krios (300 keV, Thermo Fisher) with a K3 camera (Gatan). 6,986 micrographs were collected under electron counting mode at 1.07 Å per pixel, using a defocus range of 1-2 µm with 50 electrons/Å^2 distributed into 40 fractions. The micrographs were first patch motion-corrected and contrast transfer function (CTF) estimation was performed in cryoSPARC. Particles were auto-picked by "Filament Tracer" with a shift of 20 pixels. The particles with low-resolution 2D averages and "junk" particles were removed by multiple rounds of reference-free 2D classifications. Particles having clear 2D average patterns were then selected. Typically, for amyloid and amyloid-like fibers, a strong meridional layer line is observed, which corresponds to the ~4.8 Å β-sheet separation. Using 2D classes and the average power spectrum, initial estimates of the helical pitch and the separation distances between cross-β layers were generated. However, helical reconstructions using all possible helical symmetries were unsuccessful, particularly when a mask was applied. This phenomenon has been reported and discussed in previous studies.[6] To transfer the sorted particles directly from cryoSPARC into RELION, we utilized in-house Python scripts that first mapped the sorted particles back to the filament tracer job and then converted the selected particles into a two-coordinate helical particle picking system compatible with a RELION manual picker job. This allowed us to re-extract the particles using those converted coordinates and the dose-weighted, motion-corrected images from cryoSPARC. Following an established protocol for amyloid refinement in RELION,[7, 8] we initially extracted the particles using a 320-pixel box and performed rounds of 2D classification. The helical pitch was clearly visualized in these 2D averages, remaining consistent with the estimates derived from the averaged power spectrum. A starting volume was generated using relion_helix_inimodel2d with measured pitch and used as the initial reference for the Refine3D job. After the initial 3D refinement, we performed 3D classifications with six classes. While the resulting classes did not exhibit significant morphological differences, one class demonstrated a clearly higher resolution than the other five. We then performed a subsequent Refine3D job, followed by CtfRefine and a final Refine3D job using this best class;

the final resolution of the reconstruction reached 3.3 Å, with clear cross-β separations and recognizable peptide side chains.

For peptide **4**, micrographs were collected in electron counting mode at 0.84 Å per pixel, using a defocus range of 0.6-2.5 μm and a total dose of ~50 electrons/Å^2. Data processing was performed in RELION-4.0. Motion correction and CTF estimation were done using MotionCor2 and CTFFIND4, respectively. Particles were initially picked manually, subjected to 2D classification, and used as templates for automated picking with the TOPAZ[9] filament picker. To estimate the crossover distance, particles were extracted using a large box size. Based on these estimates, multiple initial models were generated using the relion_helix_inimodel2d program and evaluated. The model exhibiting a clear separation of strands was selected for downstream processing. Particles were then re-extracted with a smaller box size, the initial model was rescaled, and iterative 3D refinement and classification steps were performed. The reconstruction successfully yielded the expected peptide backbone features with clear strand separation. During the later stages of the refinement process, the symmetry search option was enabled to optimize the helical symmetry parameters. Finally, the resulting volume was sharpened using EMReady.

Polymorphic population distributions were assessed for the four peptides from reference-free two-dimensional class averages. For each of the four systems we classified the complete set of extracted segments and quantified the population of each species from the particle counts. These analyses are presented in Figures S7 and S8. Briefly, for peptide 1, 134,016 segments were classified into 30 classes, of which classes accounting for 122,105 particles (91.1% of the dataset) correspond to the single filament morphology that was reconstructed; the remaining classes were featureless and contained no interpretable structural information. For peptide 2, 2,936,771 segments were classified into 50 classes, and the six most populated classes, comprising 2,062,959 particles (70.2%), correspond to the two polymorphs of C4 and C5 symmetry described in the manuscript. For peptide 3, classes corresponding to the reconstructed filament account for approximately 61% of the picked segments. Upon classifying 1,026,787 segments of peptide 4, the amyloid-like filament classes account for 580,442 segments, or 56.5% of the dataset, and a minor coexisting tubular species accounts for 218,638 segments, or 21.3%. Their outer widths, measured from the class averages, range from 116 to 162 Å, which indicates that the tubular species is itself

polymorphic and does not converge upon a well-defined diameter. Taken together, these analyses demonstrate that in each of the four systems the reconstructed structure corresponds to the most abundant species present under the stated assembly conditions.

Model building: For building of atomic models into the cryo-EM density of filaments derived from the bola-amphiphilic peptides, the following procedure was employed. Briefly, all reconstructions reached sub-4.0 Å resolution according to map:map FSC (Figures S9-S13) and showed clear separation of the strands along the filament axis. Since the filaments comprise β-sheets, the helical hand of the respective cryo-EM maps cannot be determined absolutely since the resolution of the reconstructions was lower than that required (≤ 2.9 Å resolution) for unambiguous assignment of the correct enantimorph. In most published cross-β structures of amyloid fibrils, the parallel β-sheet fibrils typically exhibit a left-handed helical twist. However, this assumption may not apply to filaments derived from short peptides. Therefore, we performed model building for both hands of the map. First, a peptide chain was built from the sequence in ChimeraX using the build command.[10] Peptide chains, corresponding to the subunits with the ASU, were created and manually fitted into the cryo-EM density within ChimeraX. Next, this copy of the constructed ASU and the map was opened in ISOLDE[11] and fitted into the density interactively, followed by real-space refinement in PHENIX. Helical symmetry was then applied to extend the refined ASU into 3 layers using the sym command in ChimeraX. The helically extended copies were then further refined in ISOLDE followed by real space refinement in PHENIX.[12] Final geometries of the atomic models were validated with the MolProbity[13] implementation in PHENIX. Upon comparing the models for each structure, it was found that the maps in the left-handed cross-β protofilament had either better RSCC and/or better hydrogen bonds or better model statistics. Therefore, we suggest that the maps have a left-handed twist. The refinement statistics of the respective peptide nanotubes are collected in Table S1.

For filaments derived from peptides **3** and **4**, a sequence containing a canonical phenylalanine was initially created and fitted into the cryo-EM density, as described earlier. During the later stages of refinement, this phenylalanine residue was mutated to 4-fluorophenylalanine and 3,5-difluoro-L-phenylalanine, respectively, for peptides **3** and **4**. Ligand restraints for these two non-canonical residues were generated in PHENIX using the structural CIF files obtained from the PDB ligand

database. Finally, the helical copies underwent real-space refinement in PHENIX using the generated restraint files.

Structural analysis: Least squares superposition of the peptide chains was done in PHENIX using "superpose_pdbs" command line tool or matchmaker command in ChimeraX. The pseudo-phylogenetic tree was constructed using the toytree program.[14] Lawrence-Coleman shape complementarity statistics, Sc, were calculated using the SC program in CCP4 suite using the default parameters.[15] As the program did not recognize the ligands, the library file was modified to include their van der Waals radii prior to calculations. The buried surface area was calculated using the "measure buriedarea" command in ChimeraX. We selected 3 rungs for calculating the buried area. The value thus obtained was divided by the number of rungs to get a better approximation of the buried area.[16] The relative solvent accessible surface area (rSASA) was estimated for the phenylalanine derivatives using the values from Tien, et al.[17] For the non-canonical 4-fluorophenylalanine and 3,5-difluorophenylalanine derivatives, the empirical value for the near isostere tyrosine was employed to estimate the rSASA values. Solvation free energy calculations were performed using the Fortran-based Amyloid Illustrator scripts made available online[18] (https://zenodo.org/records/15218932). Since the non-canonical amino acids 4-fluorophenylalanine and 3,5-difluorophenylalanine were not recognized by the program for calculating the solvation free energy parameters, another input was created in which these residues were substituted with phenylalanine.

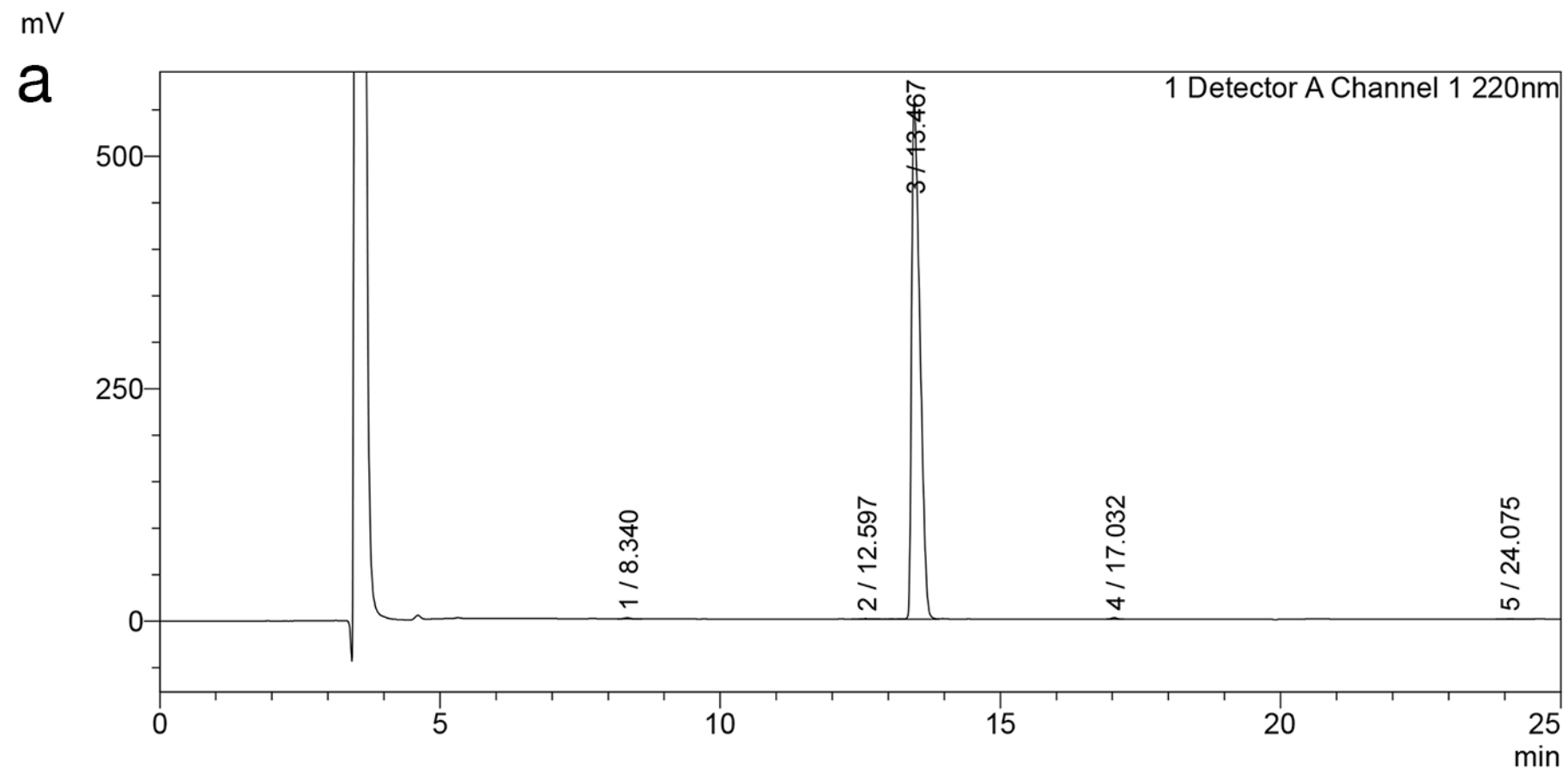

<Peak Table>

Detector A Channel 1 220nm

Peak#	Ret. Time	Area	Height	Area%
1	8.340	8938	1123	0.155
2	12.597	10765	637	0.187
3	13.467	5730561	552382	99.344
4	17.032	9571	1393	0.166
5	24.075	8582	383	0.149
Total		5768418	555918	100.000

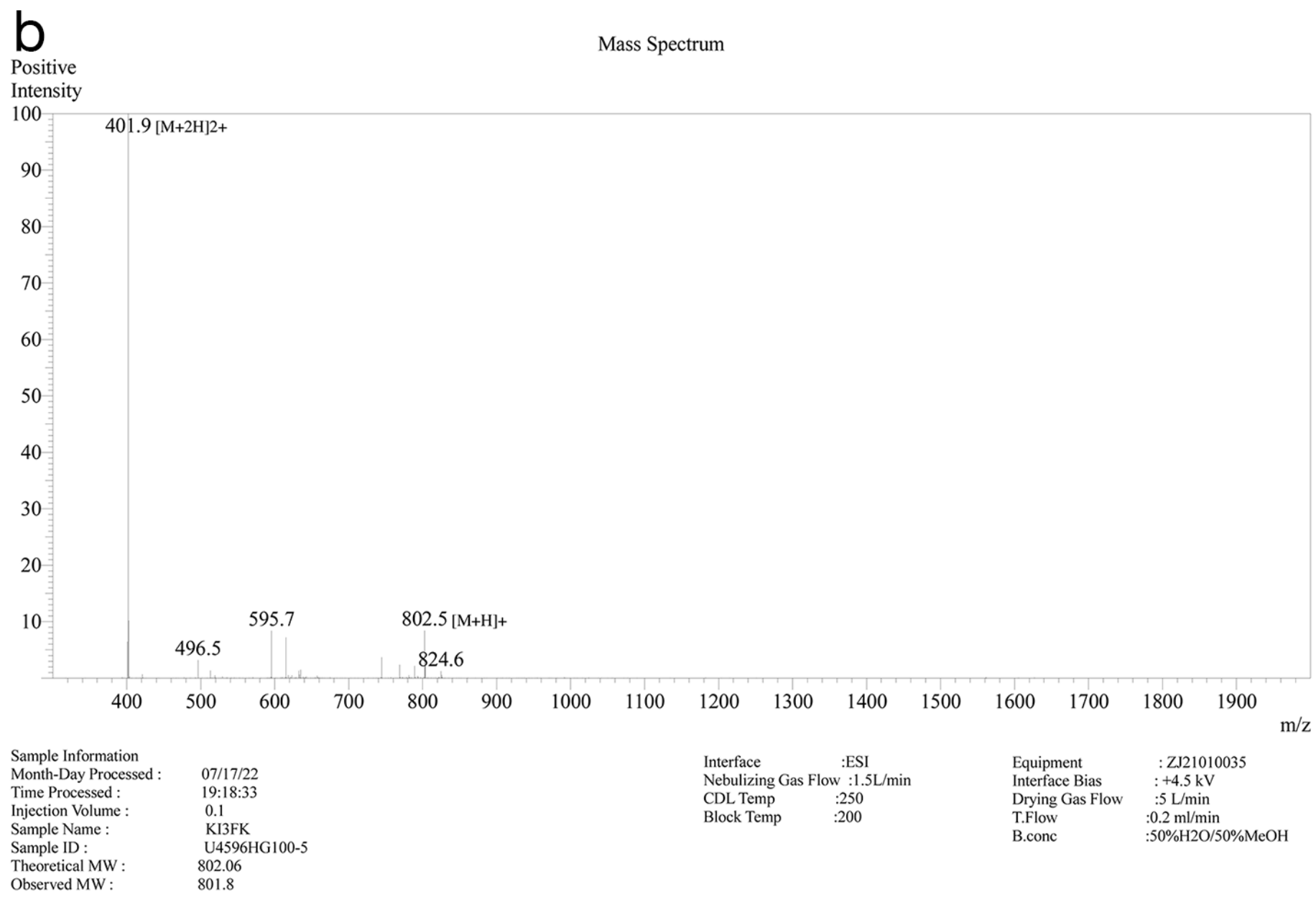

Figure S1. Analytical HPLC (**a**) and ESI-MS (**b**) data for peptide **1**, Ac-Lys-Ile-Ile-Ile-Phe-Lys-NH_2. Peptide purity was estimated at 99.3%. Calculated monoisotopic mass for peptide **1**, 801.5 Da; experimental mass from mass spectrum, 802.5 Da.

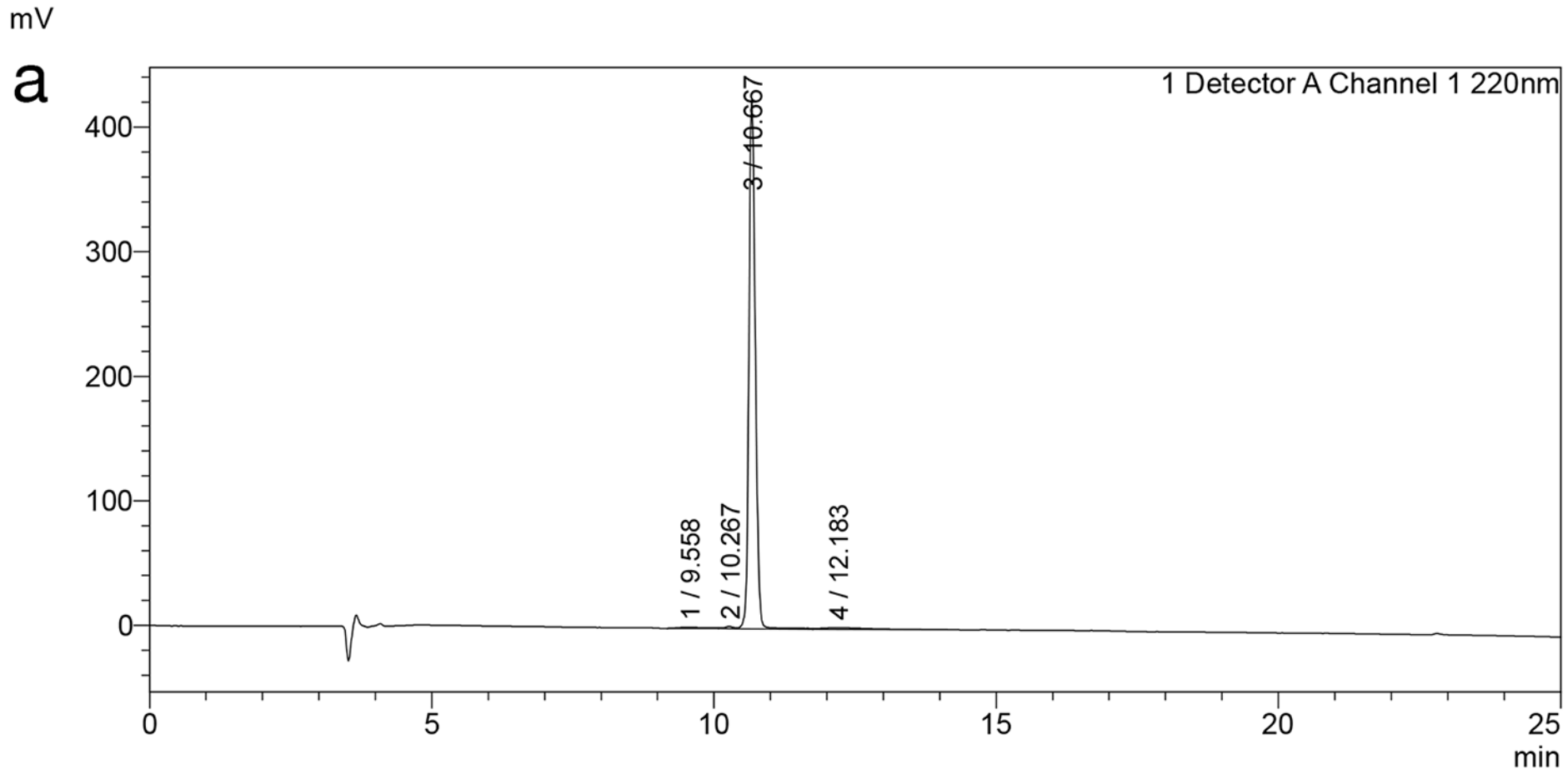

<Peak Table>

Detector A Channel 1 220nm

Peak#	Ret. Time	Area	Height	Area%
1	9.558	37294	1005	1.066
2	10.267	20906	1760	0.598
3	10.667	3363884	425431	96.171
4	12.183	75726	1350	2.165
Total		3497809	429547	100.000

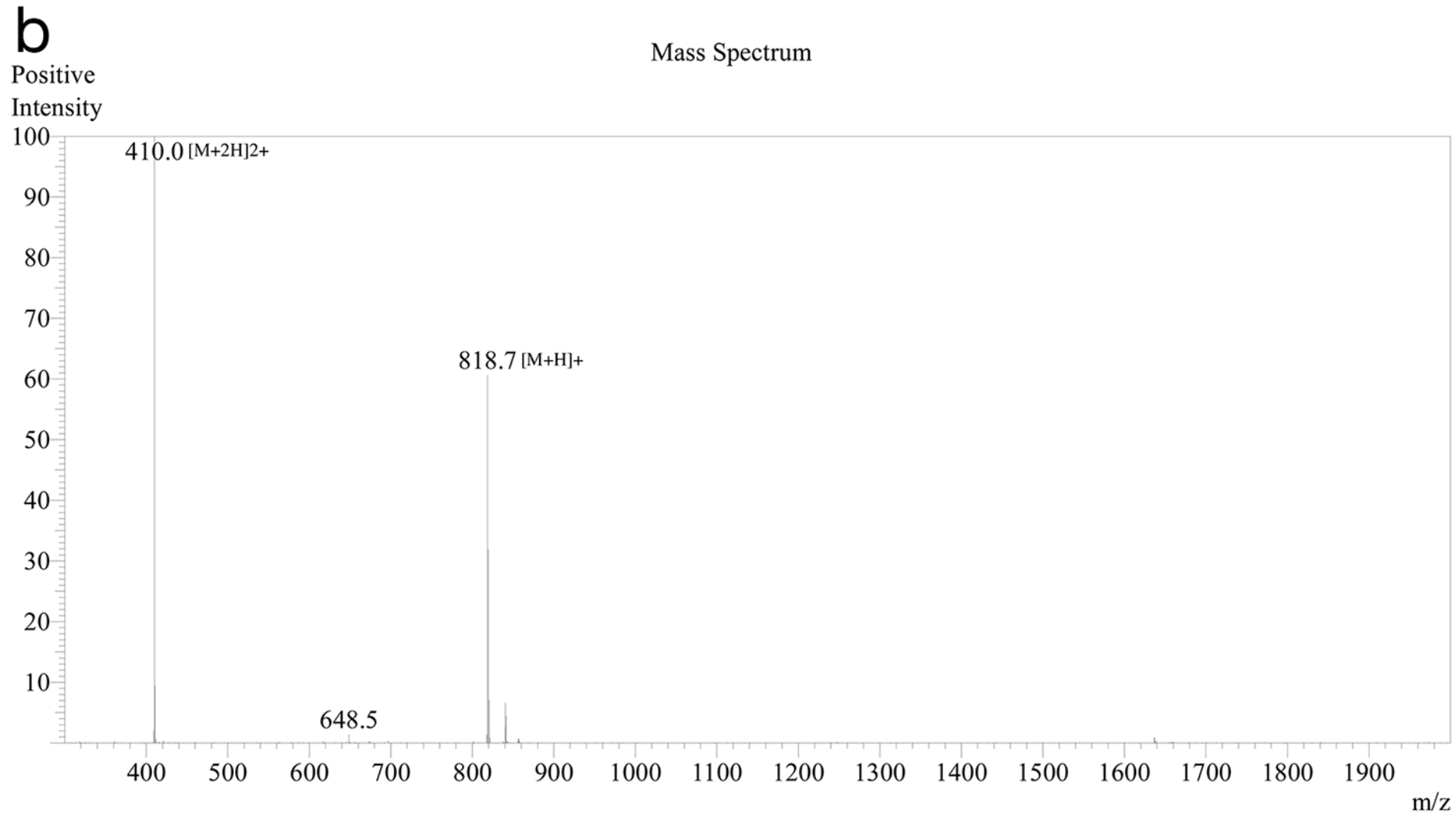

Sample Information
Month-Day Processed : 12/15/24
Time Processed : 15:28:23
Injection Volume : 0.5
Sample Name : Y5
Sample ID : U524C341G0-1
Theoretical MW : 818.07
Observed MW : 818.0

Interface :ESI
Nebulizing Gas Flow :1.5L/min
CDL Temp :250
Block Temp :200

Equipment : ZJ21010035
Interface Bias : +4.5 kV
Drying Gas Flow :5 L/min
T.Flow :0.2 ml/min
B.conc :50%H2O/50%MeC

Figure S2. Analytical HPLC (**a**) and ESI-MS (**b**) data for peptide **2**, Ac-Lys-Ile-Ile-Ile-Tyr-Lys-NH_2. Peptide purity was estimated at 96.2%. Calculated monoisotopic mass for peptide **2**, 817.5 Da; experimental mass from mass spectrum, 818.7 Da.

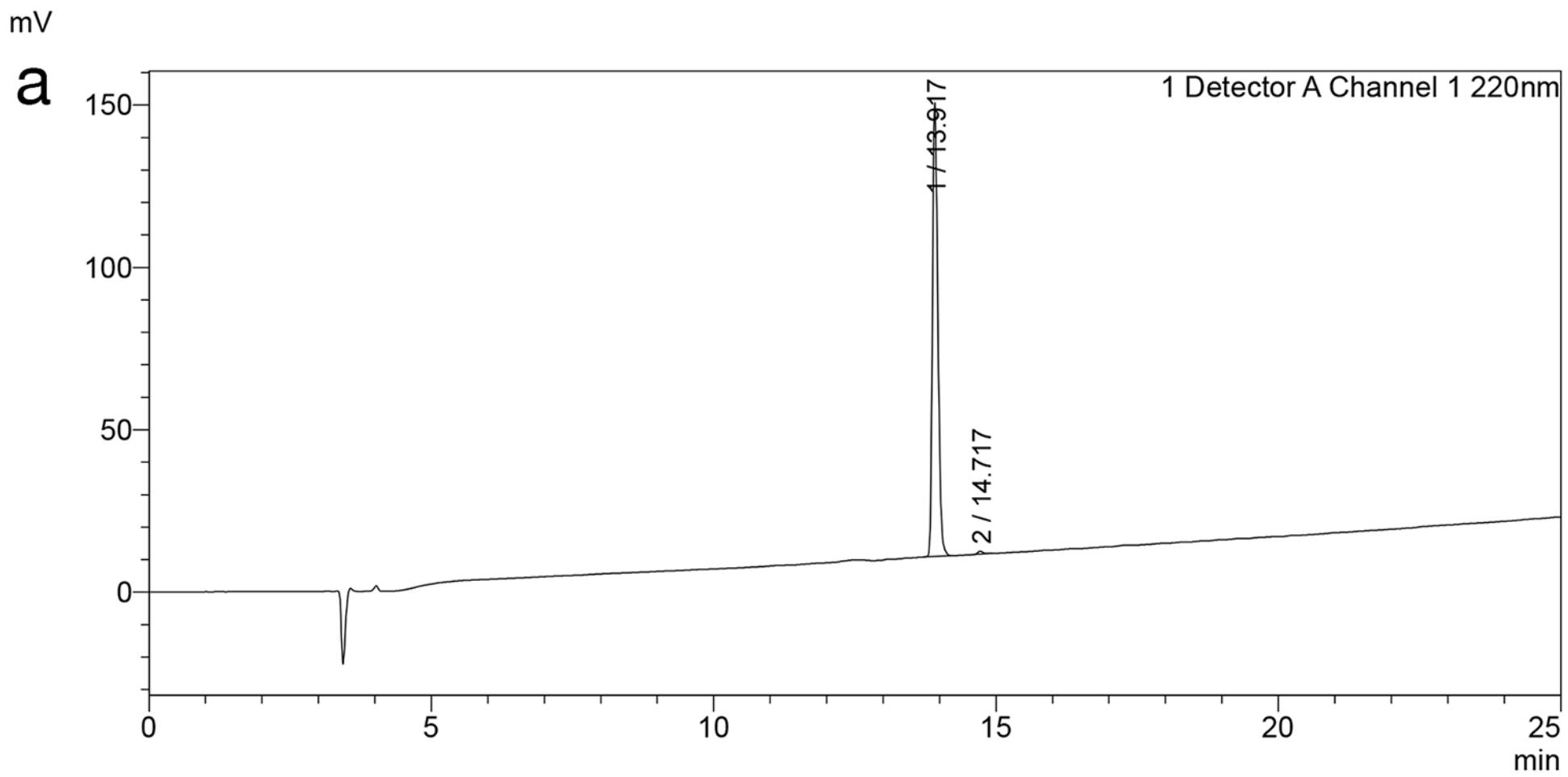

<Peak Table>

Detector A Channel 1 220nm

Peak#	Ret. Time	Area	Height	Area%
1	13.917	936290	139851	99.423
2	14.717	5429	862	0.577
Total		941719	140713	100.000

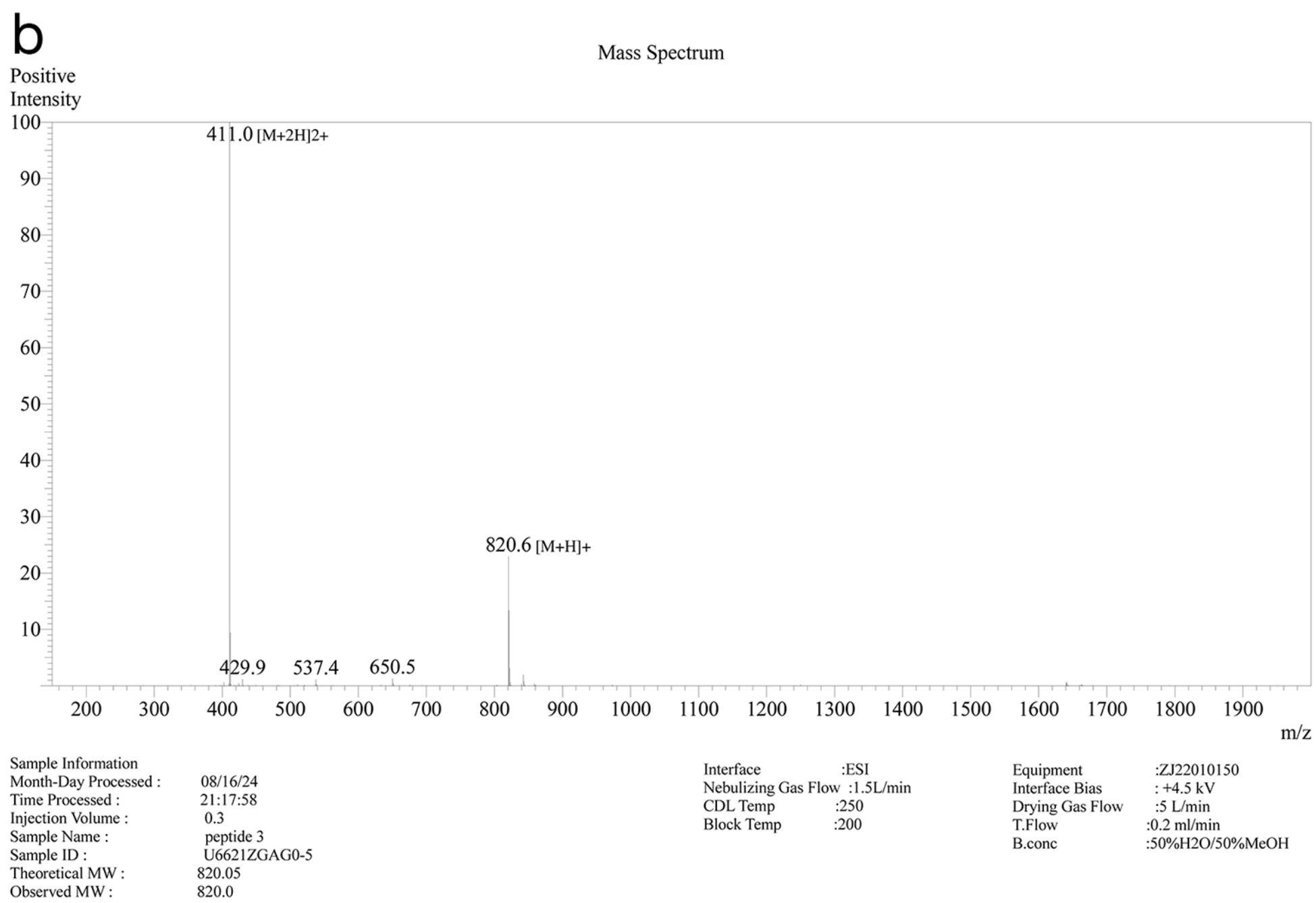

Figure S3. Analytical HPLC (**a**) and ESI-MS (**b**) data for peptide **3**, Ac-Lys-Ile-Ile-Ile-(4F-Phe)-Lys-NH_2. Peptide purity was estimated at 99.4%. Calculated monoisotopic mass for peptide **3**, 819.5 Da; experimental mass from mass spectrum, 820.6 Da.

a

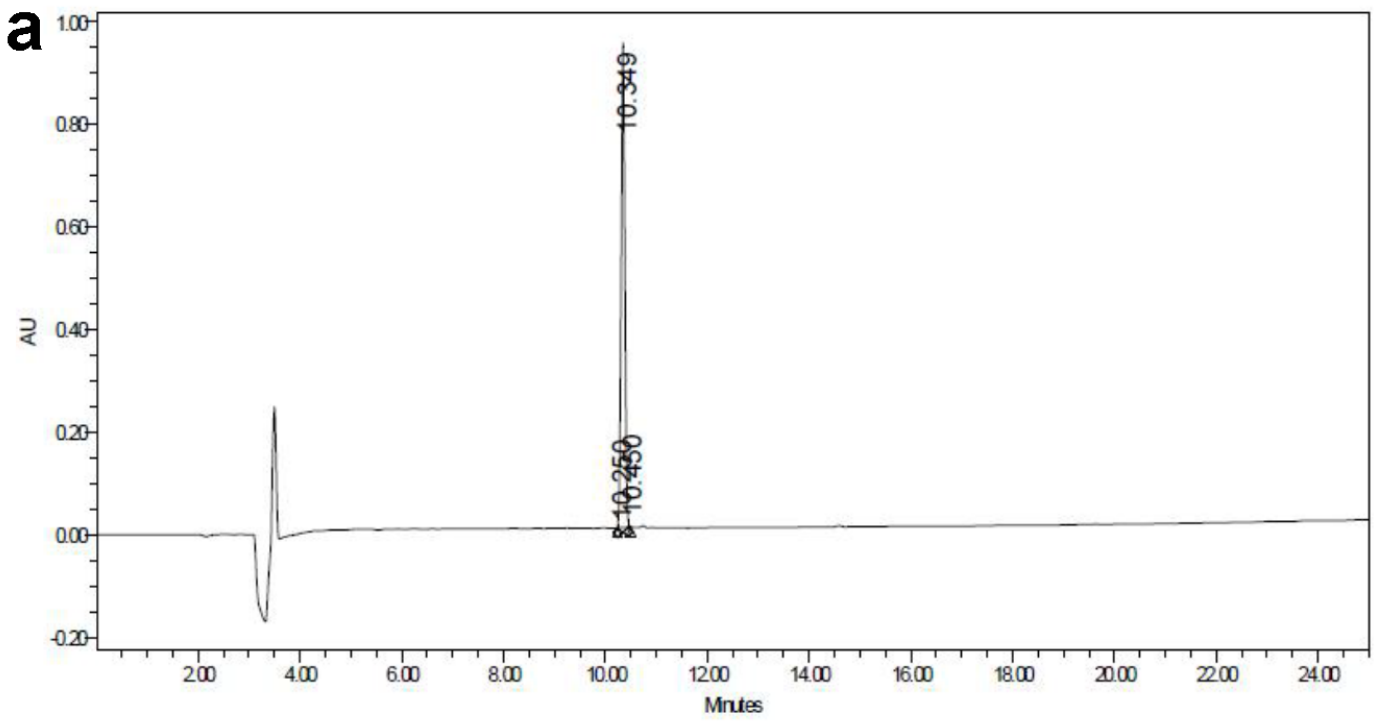

	RT	Area	%Area	Height
1	10.250	3041	0.07	6081
2	10.349	4594813	99.64	930407
3	10.450	13767	0.30	14911

b

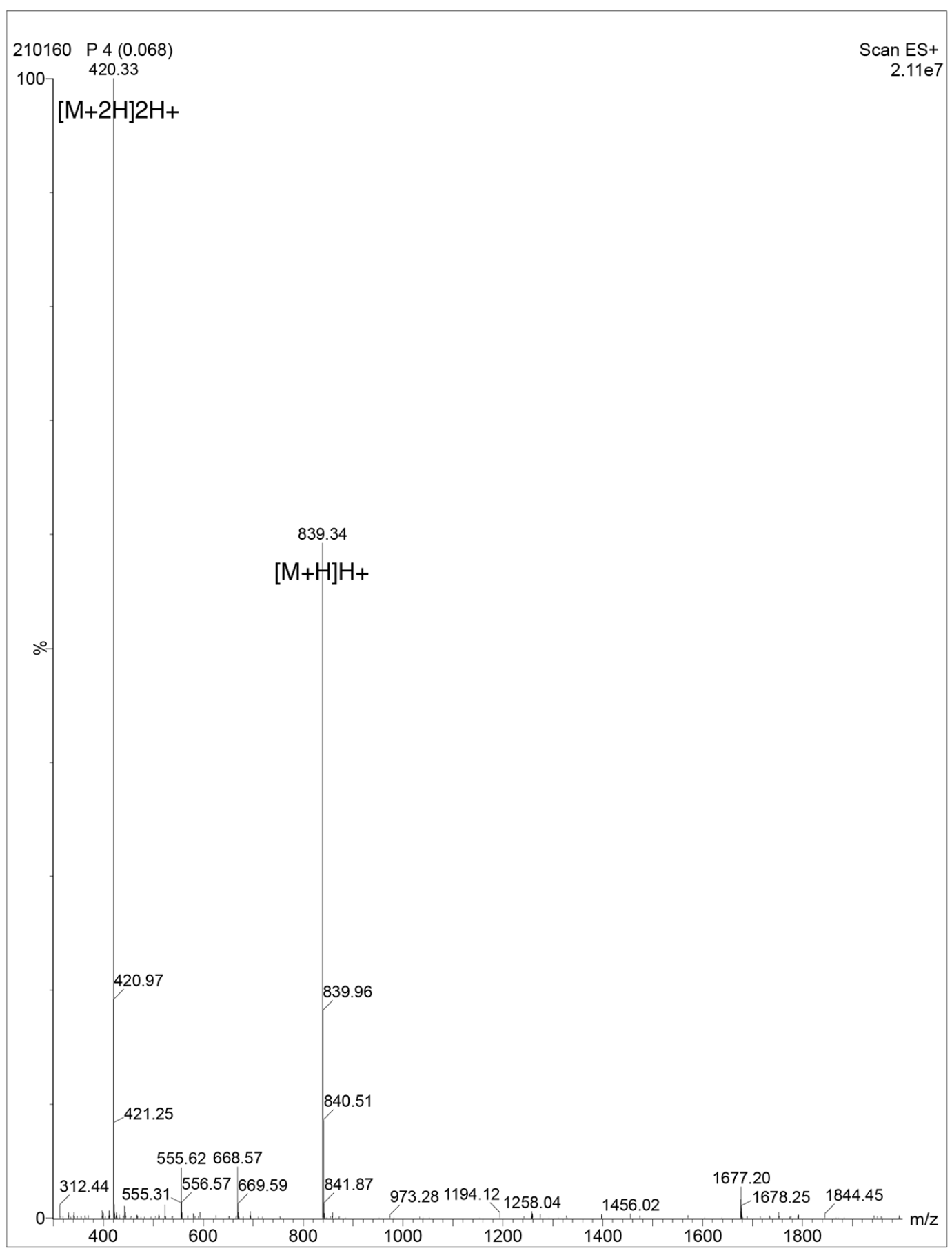

Figure S4. Analytical HPLC (**a**) and ESI-MS (**b**) data for peptide **4**, Ac-Lys-Ile-Ile-Ile-(3,5F-Phe)-Lys-NH_2. Peptide purity was estimated at 99.64%. Calculated monoisotopic mass for peptide **4**, 838.3 Da; experimental mass from mass spectrum, 839.3 Da.

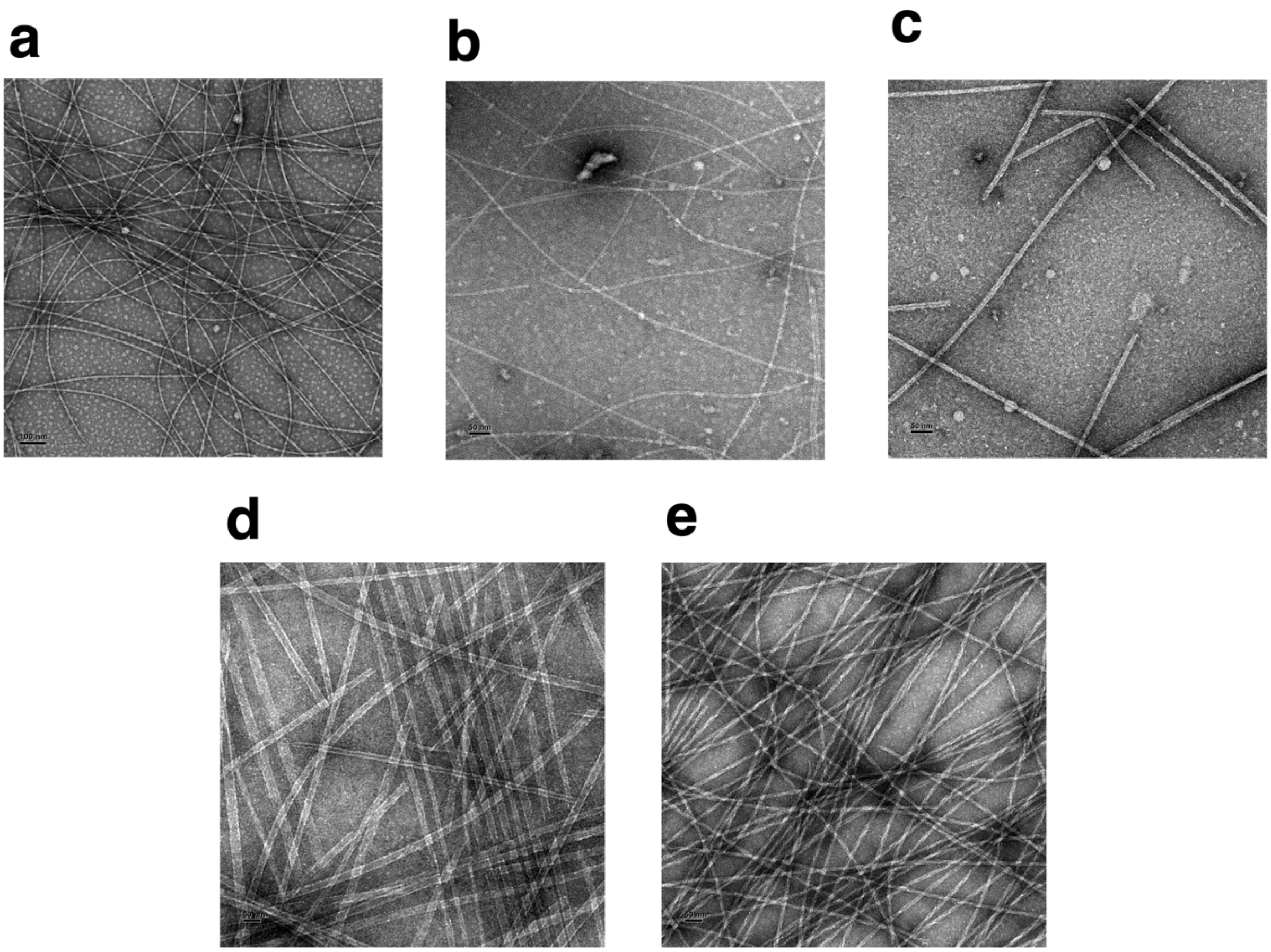

Figure S5. Representative negative stain TEM micrographs of peptide **1** (**a**), peptide **2** (**b**), peptide **3** (**c**), and peptide **4** at ambient temperature (**d**) and after thermal annealing (**e**). Prior to deposition on EM grids for image acquisition, the peptide stock solutions (~32 mM) were diluted 10-fold.

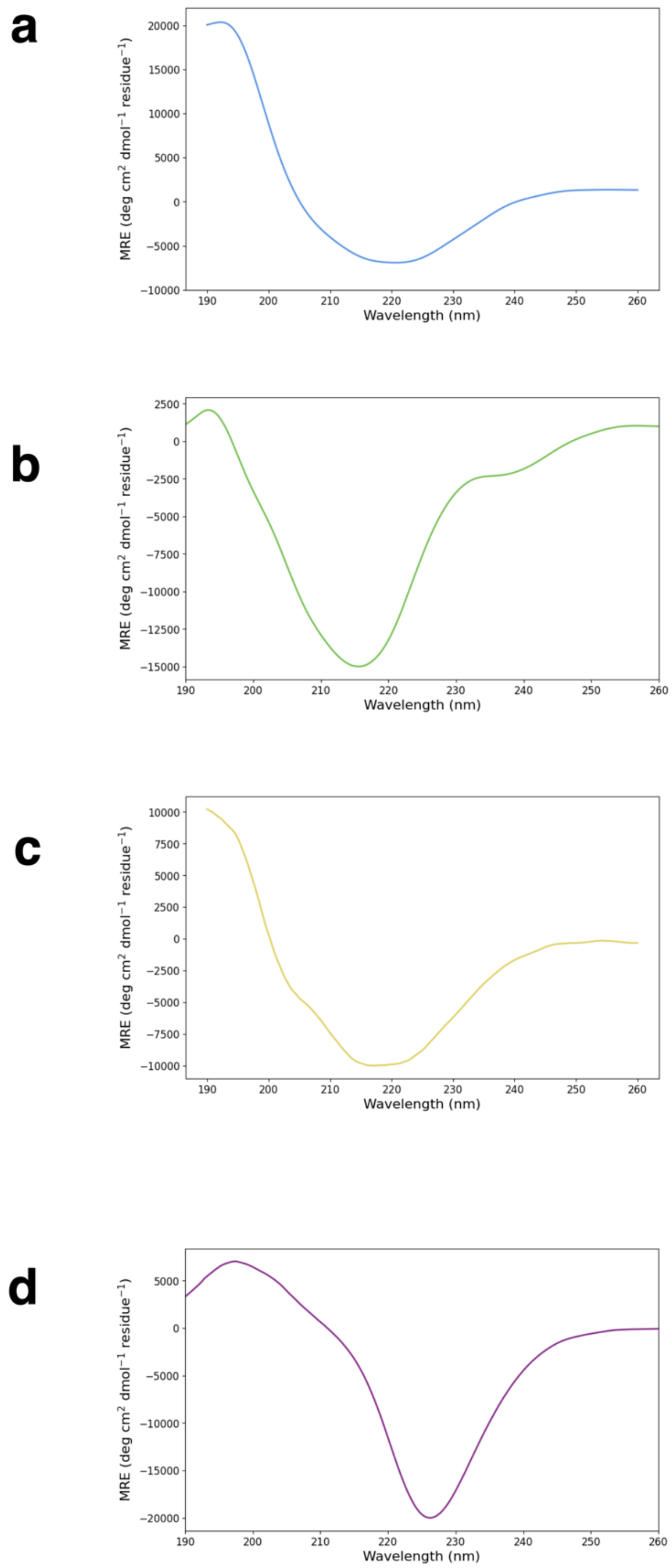

Figure S6. CD spectra of peptide **1** (**a**), peptide **2** (**b**), peptide **3** (**c**), and peptide **4** (**d**) displaying minima near 220 nm consistent with formation of β-sheet. The red shift is likely due to aromatic side chain contribution to the far UV region.

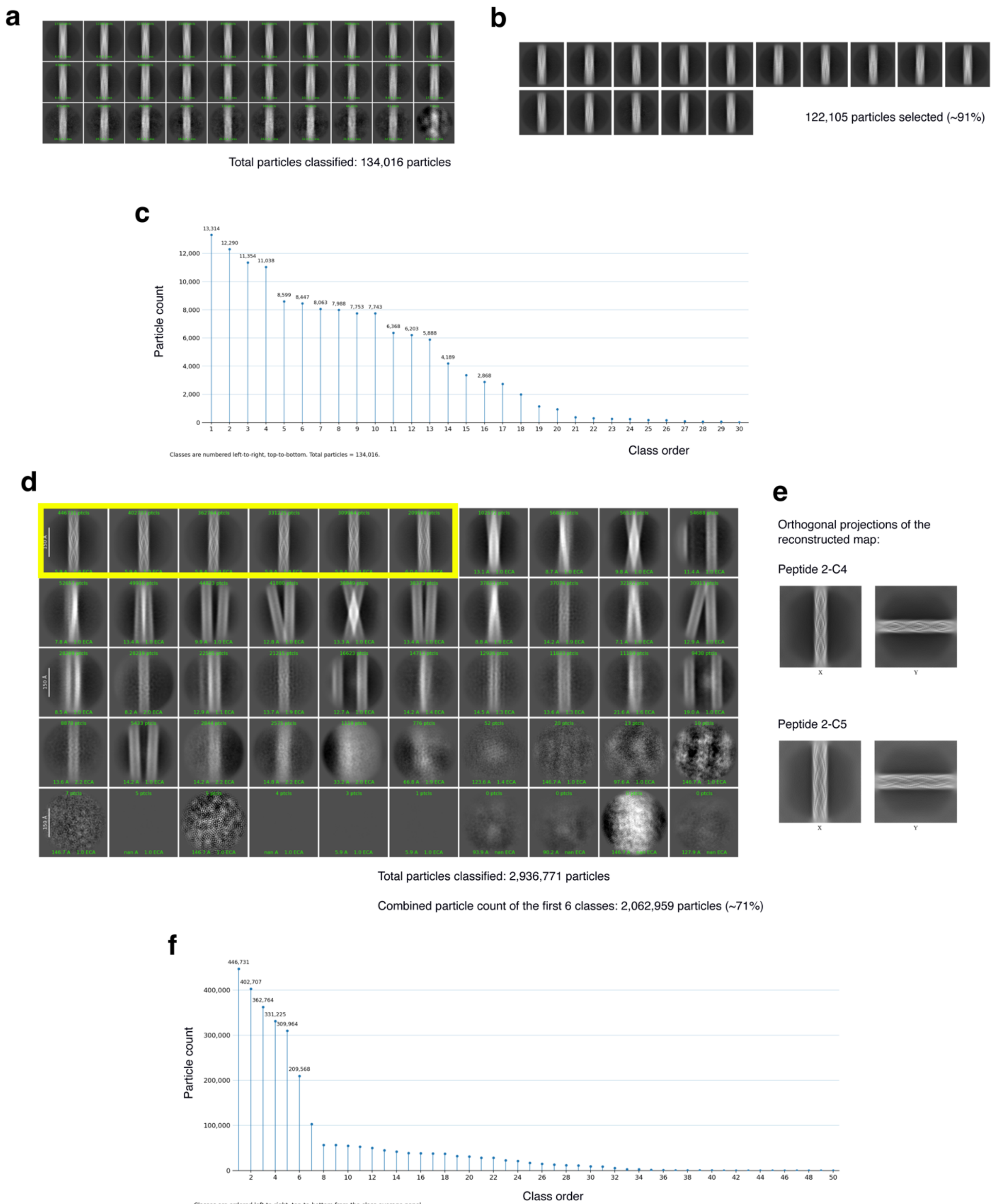

Figure S7. (**a**) Reference-free 2D class averages of peptide **1**, from 134,016 particles classified into 30 classes. (**b**) Classes retained after selection, comprising 122,105 particles (~91% of the total). (**c**) Histogram of particle count per class for the classification in (**a**), ordered by population; classes are numbered left-to-right, top-to-bottom. (**d**) Reference-free 2D class averages of peptide

2, from 2,936,771 particles classified into 50 classes. The six most populated classes (yellow box) together account for 2,062,959 particles (~71% of the total) and correspond to the two reconstructed polymorphs. (**e**) Orthogonal projections of the reconstructed maps for the C4 and C5 polymorphs of peptide **2**, shown for comparison with the class averages in (**d**). (**f**) Histogram of particle count per class for the classification in (**d**). In both systems, the classes corresponding to the reconstructed structures represent the majority of the particle population, indicating that the solved structures are the predominant species under these assembly conditions rather than minor polymorphs.

a

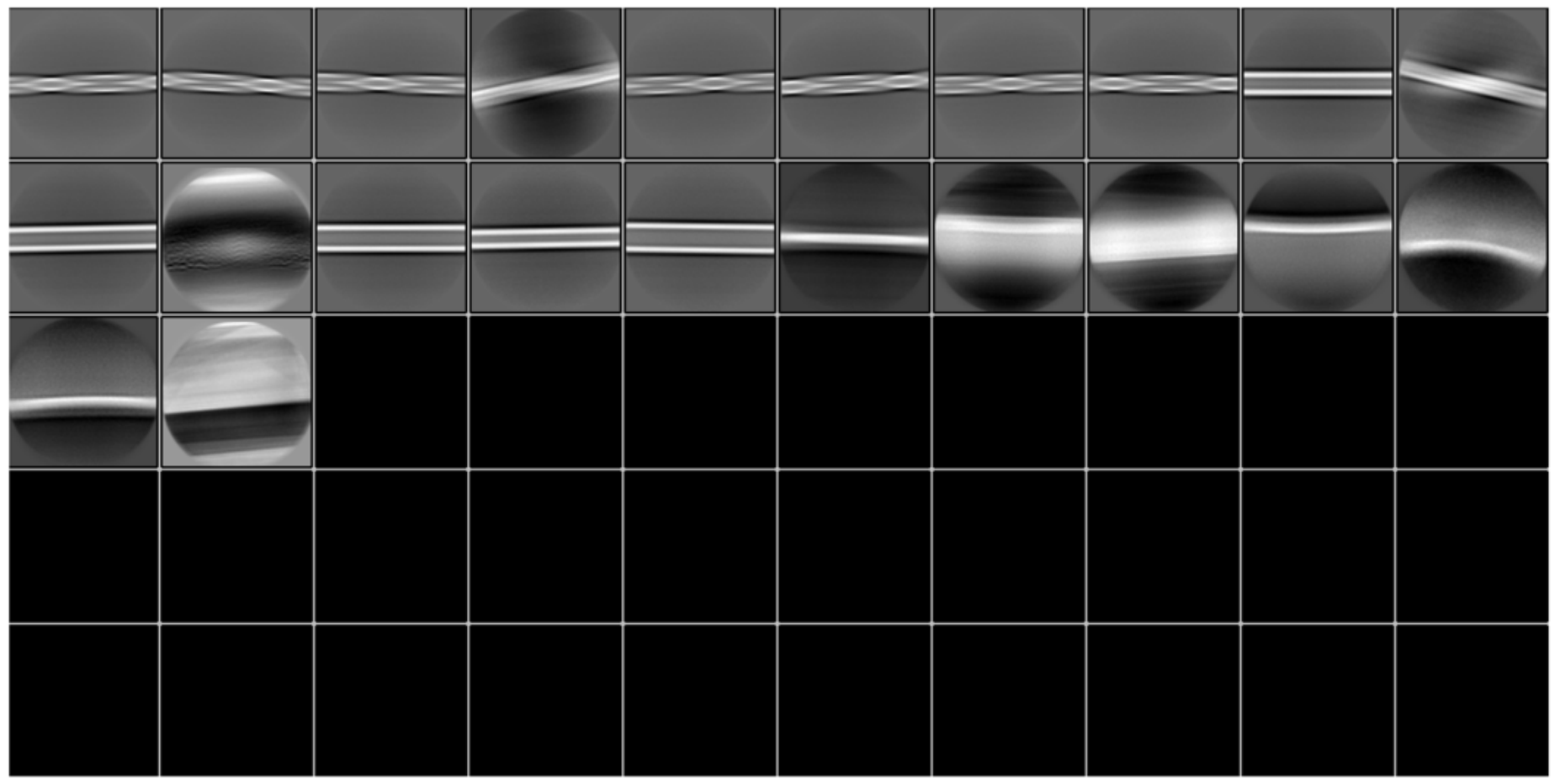

b

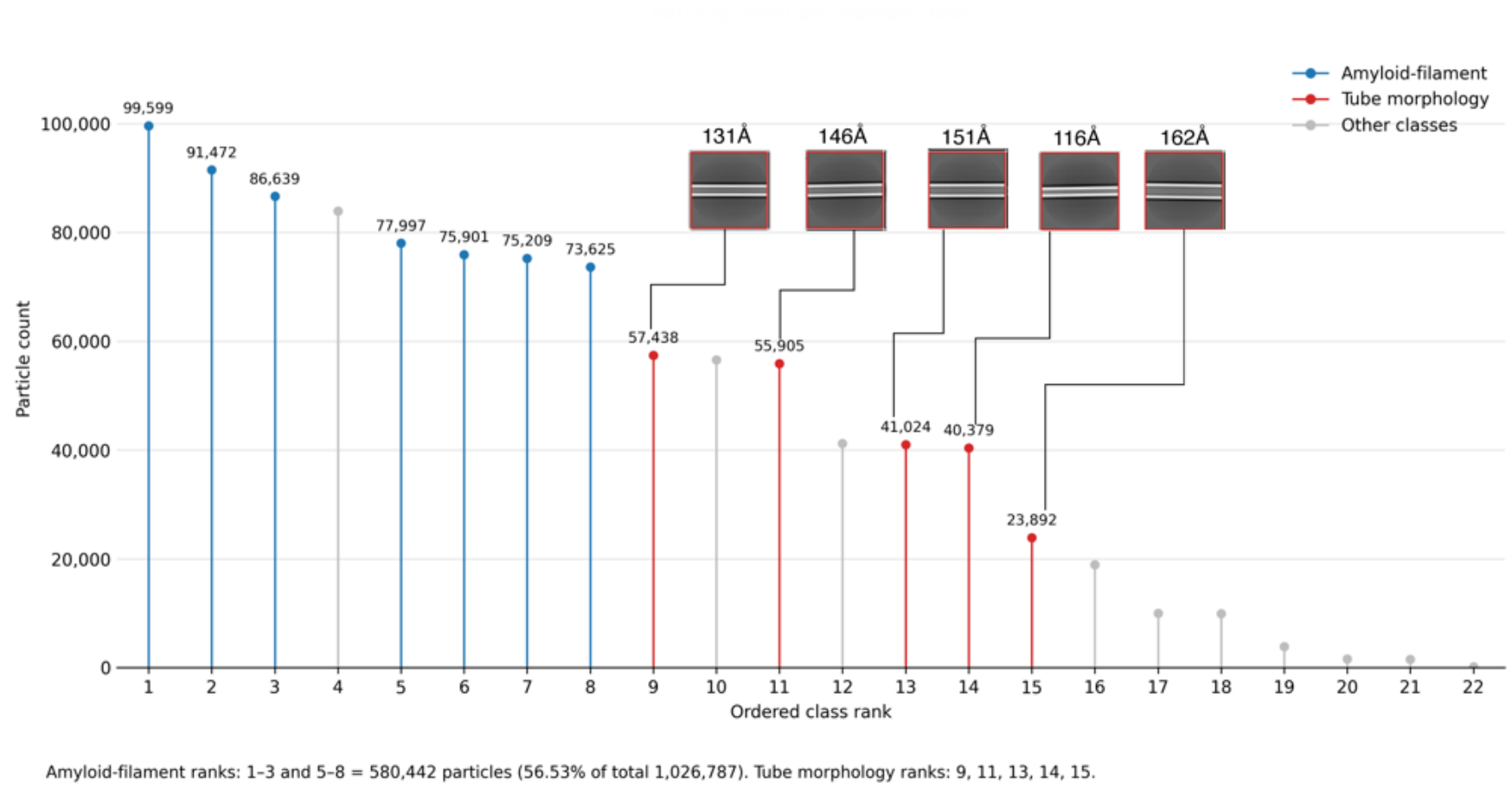
Amyloid-filament
Tube morphology
Other classes
131Å
146Å
151Å
116Å
162Å
99,599
91,472
86,639
77,997
75,901
75,209
73,625
57,438
55,905
41,024
40,379
23,892
Particle count
100,000
80,000
60,000
40,000
20,000
0
1
2
3
4
5
6
7
8
9
10
11
12
13
14
15
16
17
18
19
20
21
22
Ordered class rank
Amyloid-filament ranks: 1–3 and 5–8 = 580,442 particles (56.53% of total 1,026,787). Tube morphology ranks: 9, 11, 13, 14, 15.

c

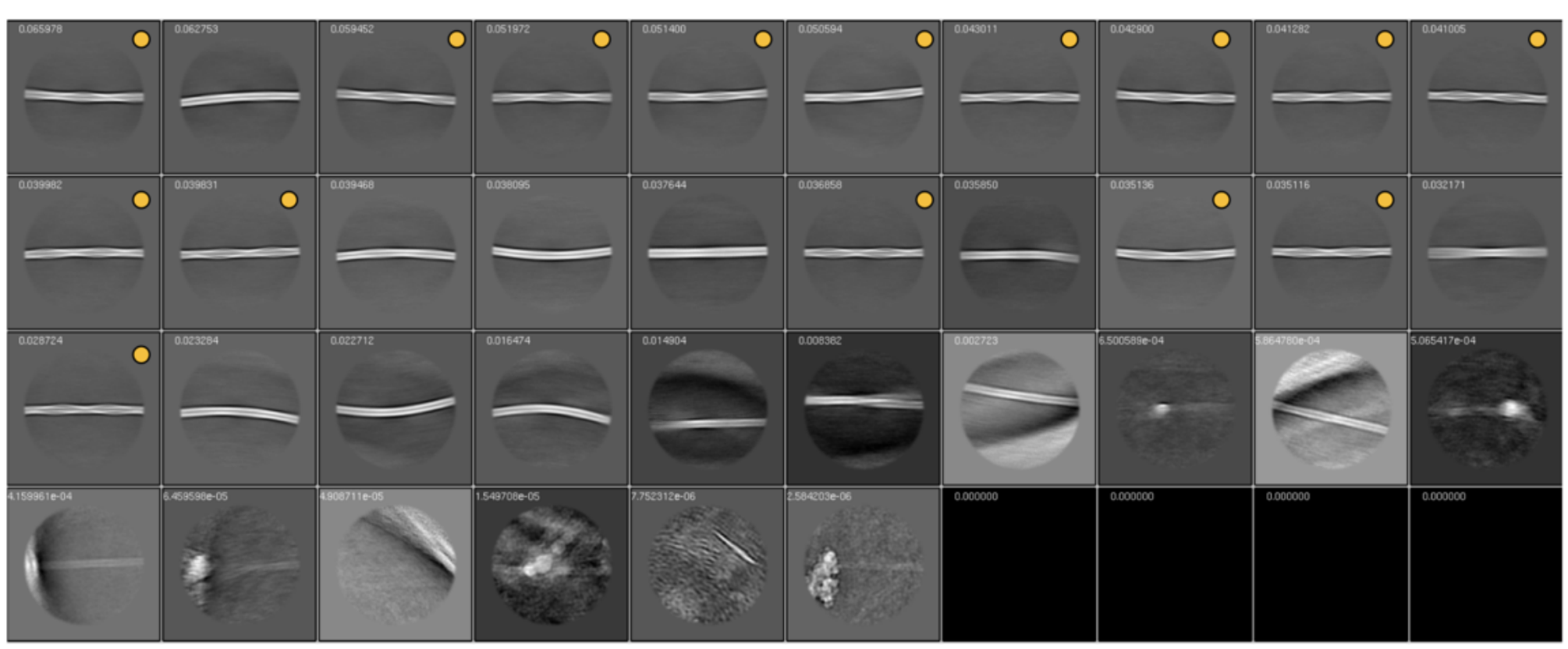

Figure S8. (**a**) Reference-free 2D class averages obtained from the full set of extracted segments for peptide **4**. The majority of classes display the cross-section characteristic of the amyloid-like filament. A smaller number of classes show hollow tubular morphology, together with a subset of poorly resolved or featureless classes. (**b**) Particle-count distribution across the 22 classes, ordered by decreasing occupancy. Bars are colored by the morphology assigned to each class average: amyloid-filament (blue), tube (red), and unassigned or low-quality classes (grey). Classes assigned to the tubular morphology (ranks 9, 11, 13, 14, and 15) are shown as insets, with the outer width measured from the class average indicated above each (116–162 Å). The amyloid-filament classes (ranks 1–3 and 5–8) account for 580,442 of 1,026,787 segments (56.5% of the dataset), whereas the tube classes together account for 218,638 segments (21.3%). The heterogeneity in measured tube width across these classes indicates that the tubular species is itself polymorphic and does not converge on a single well-defined diameter. Tube classes were attempted for helical reconstruction and failed to converge. (**c**) Reference-free 2D classification of the extracted particles for peptide **3**, with class averages ordered by decreasing occupancy and the corresponding class distribution given at the upper left of each. Yellow dots denote the classes selected at the outset for downstream processing, accounting for ~61% of the total particle population; the remaining ~39% populate classes devoid of interpretable features and were discarded.

a

b

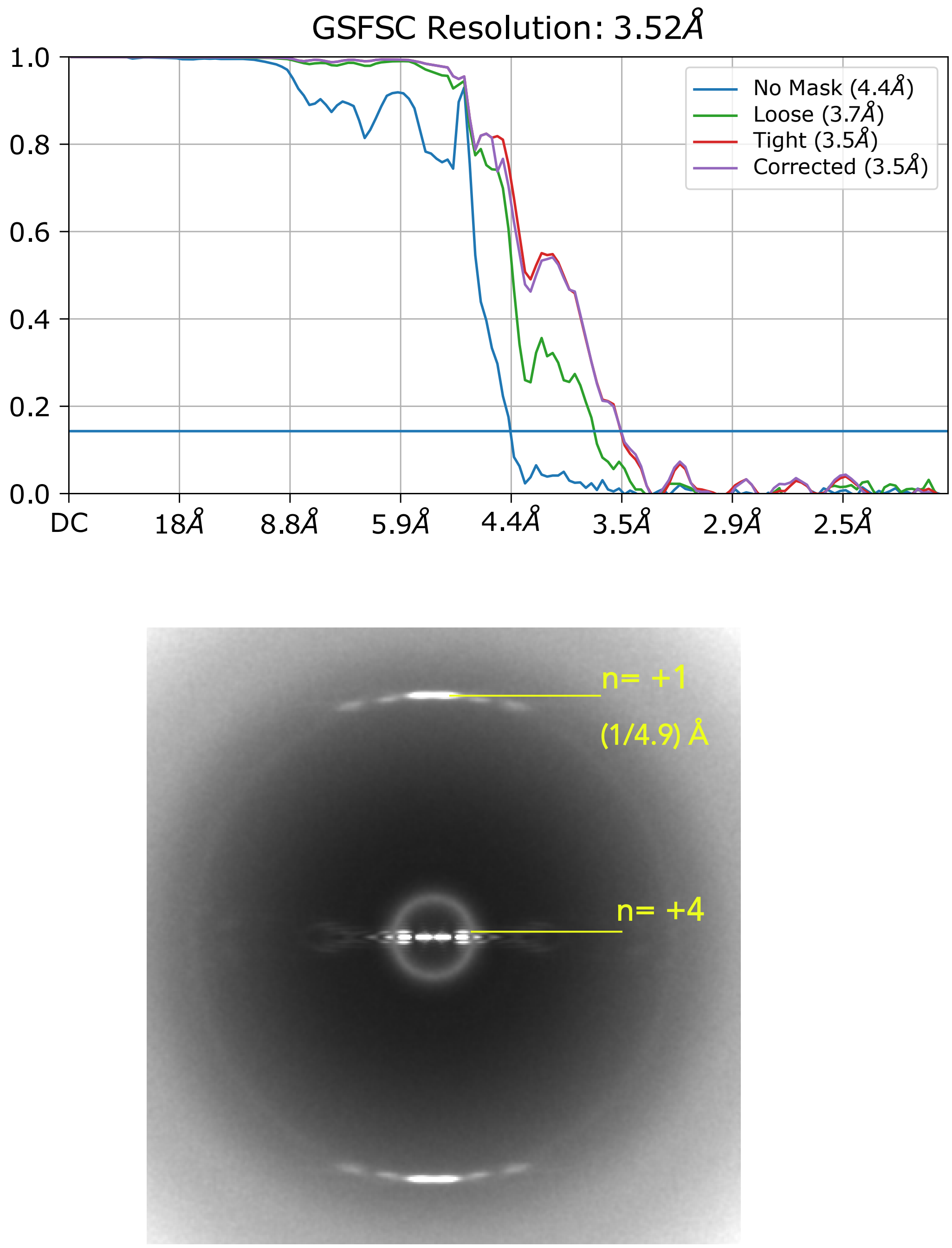

Figure S9. (**a**) Gold-standard map:map Fourier Shell Correlation (FSC) calculations for peptide **1** filament (0.143 criterion). (**b**) Averaged power spectrum of the particles used in the initial reconstruction showing the indexed layer-lines and the cross-β characteristic layer-line at 1/4.9 Å

a

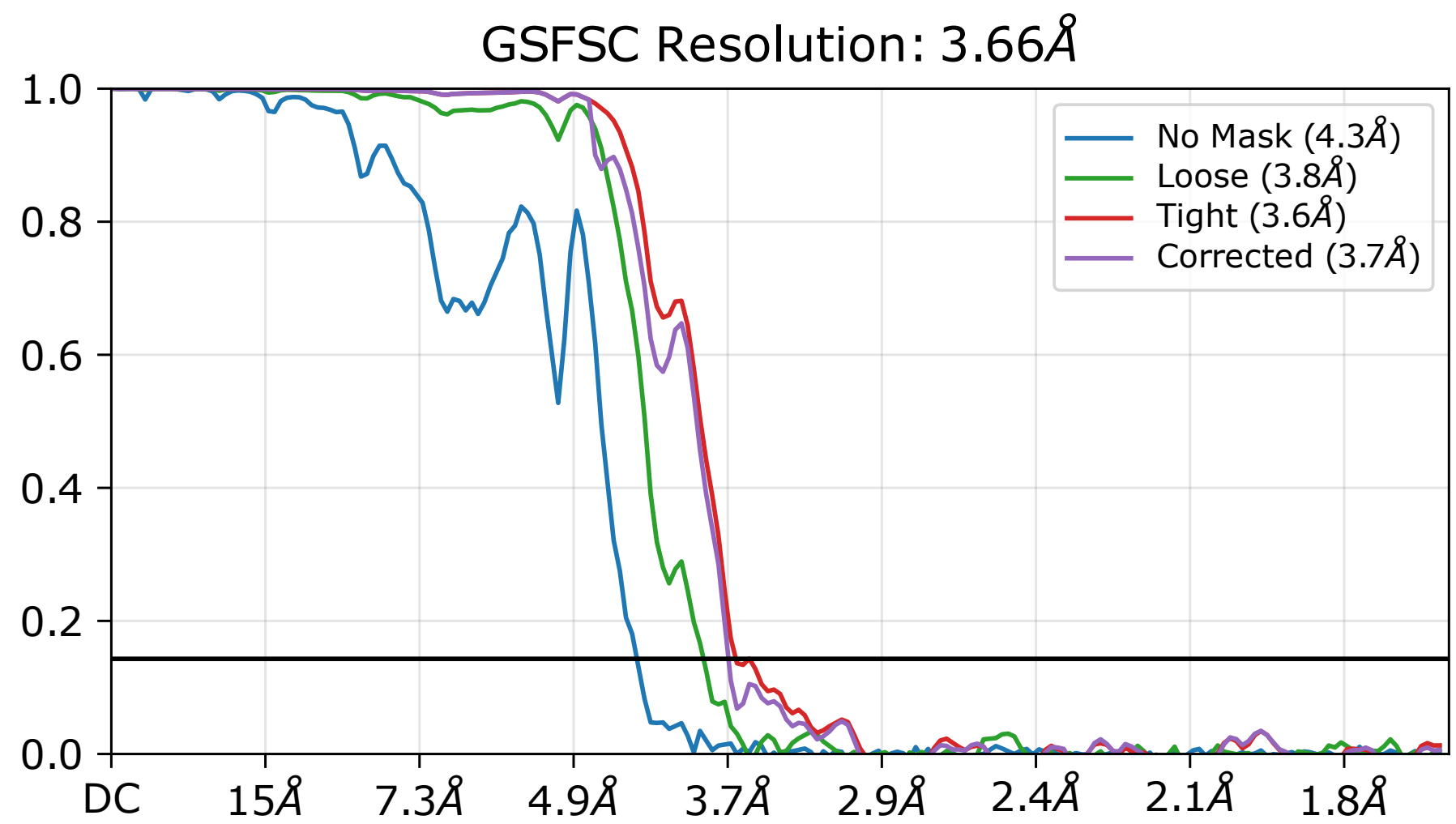

b

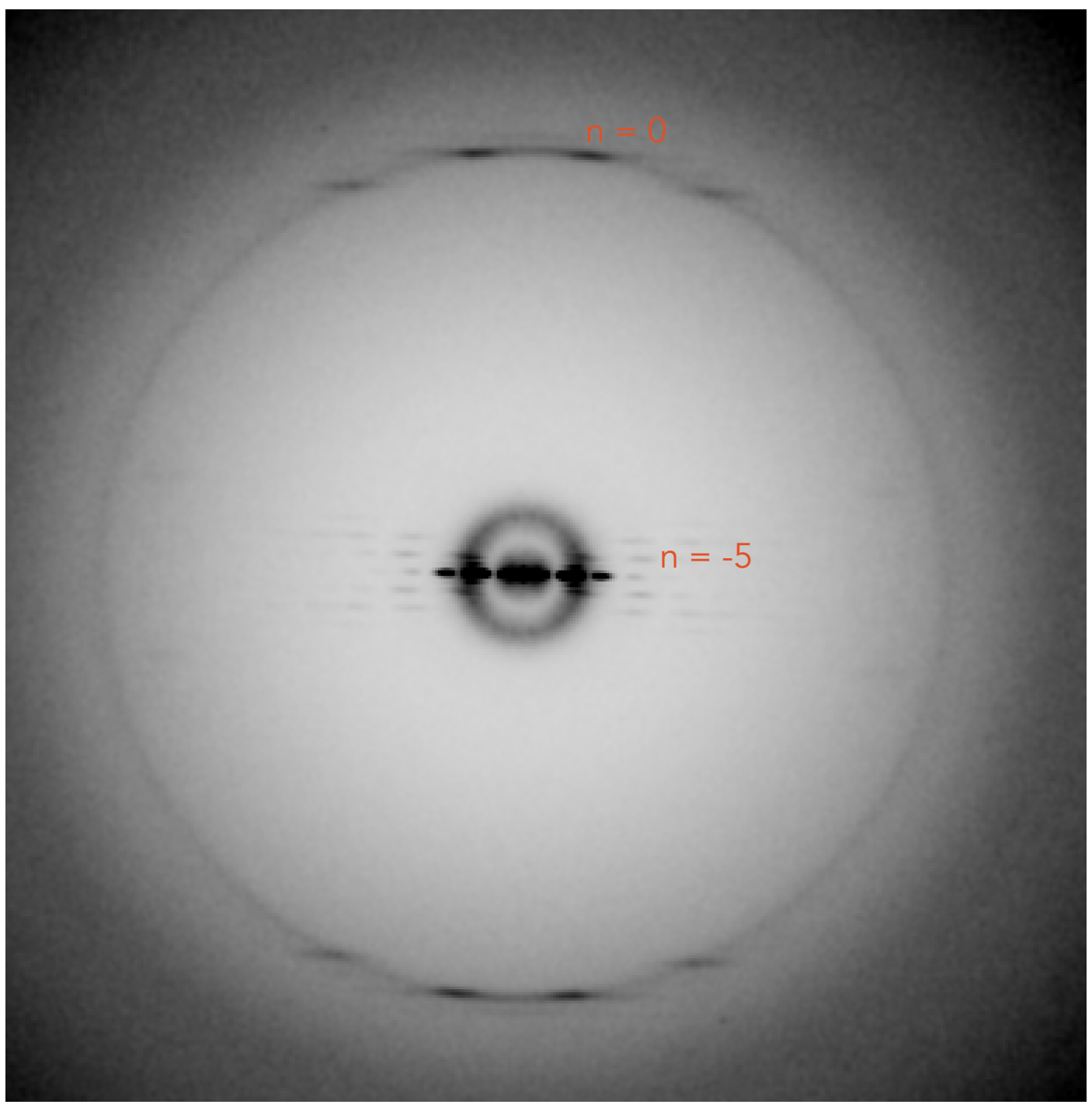

Figure S10. (**a**) Gold-standard map:map Fourier Shell Correlation (GS-FSC) calculations for the peptide **2**-C5 filament (0.143 criterion). (**b**) Averaged power spectrum of the particles used in the initial reconstruction showing the indexed layer-lines.

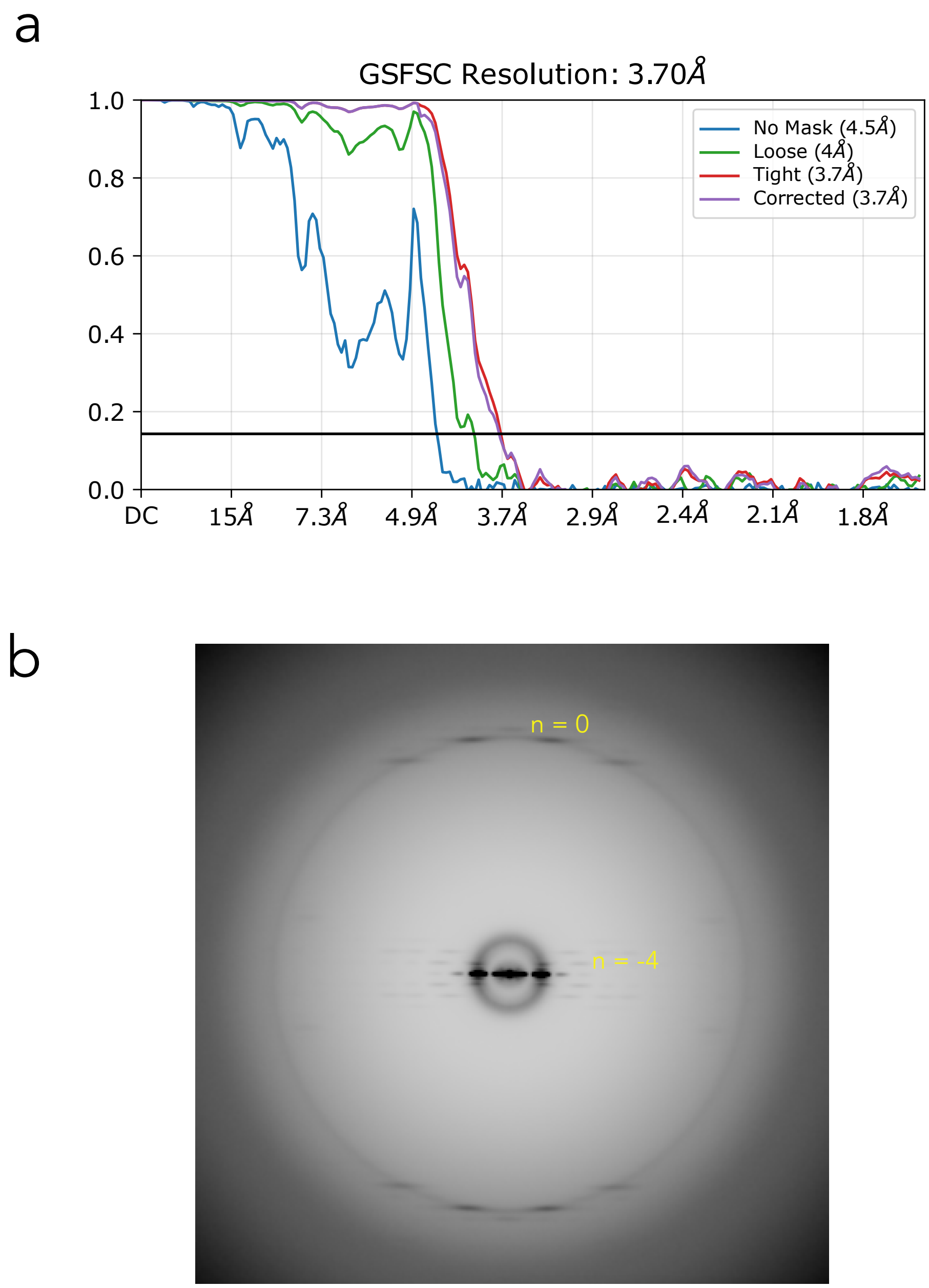

Figure S11. (**a**) Gold-standard map:map Fourier Shell Correlation (GS-FSC) calculations for peptide **2**-C4 filament (0.143 criterion). (**b**) Averaged power spectrum of the particles used in the initial reconstruction showing the indexed layer-lines.

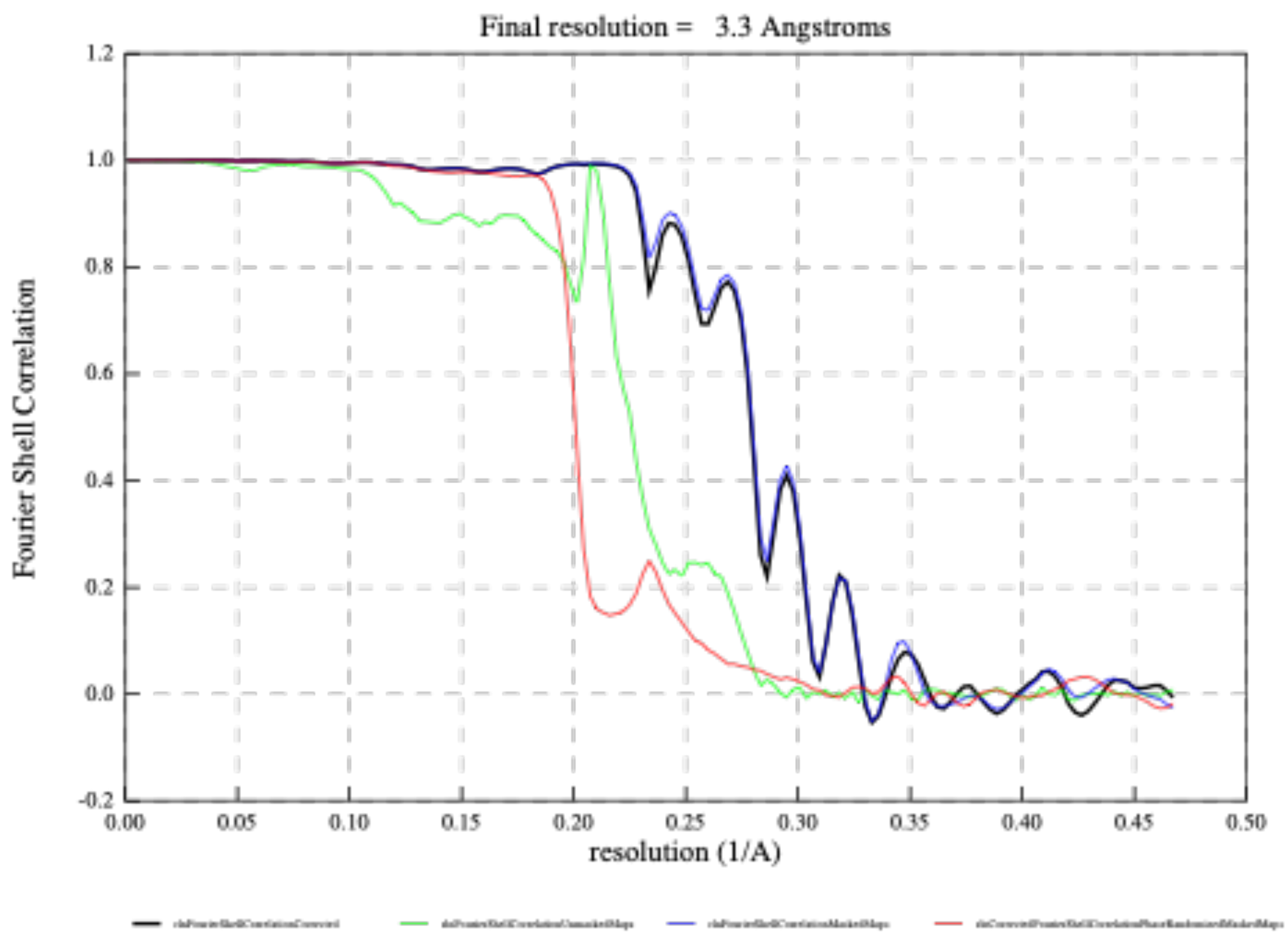

Figure S12. Gold-standard map:map Fourier Shell Correlation (GS-FSC) calculations for peptide **3** filament (0.143 criterion).

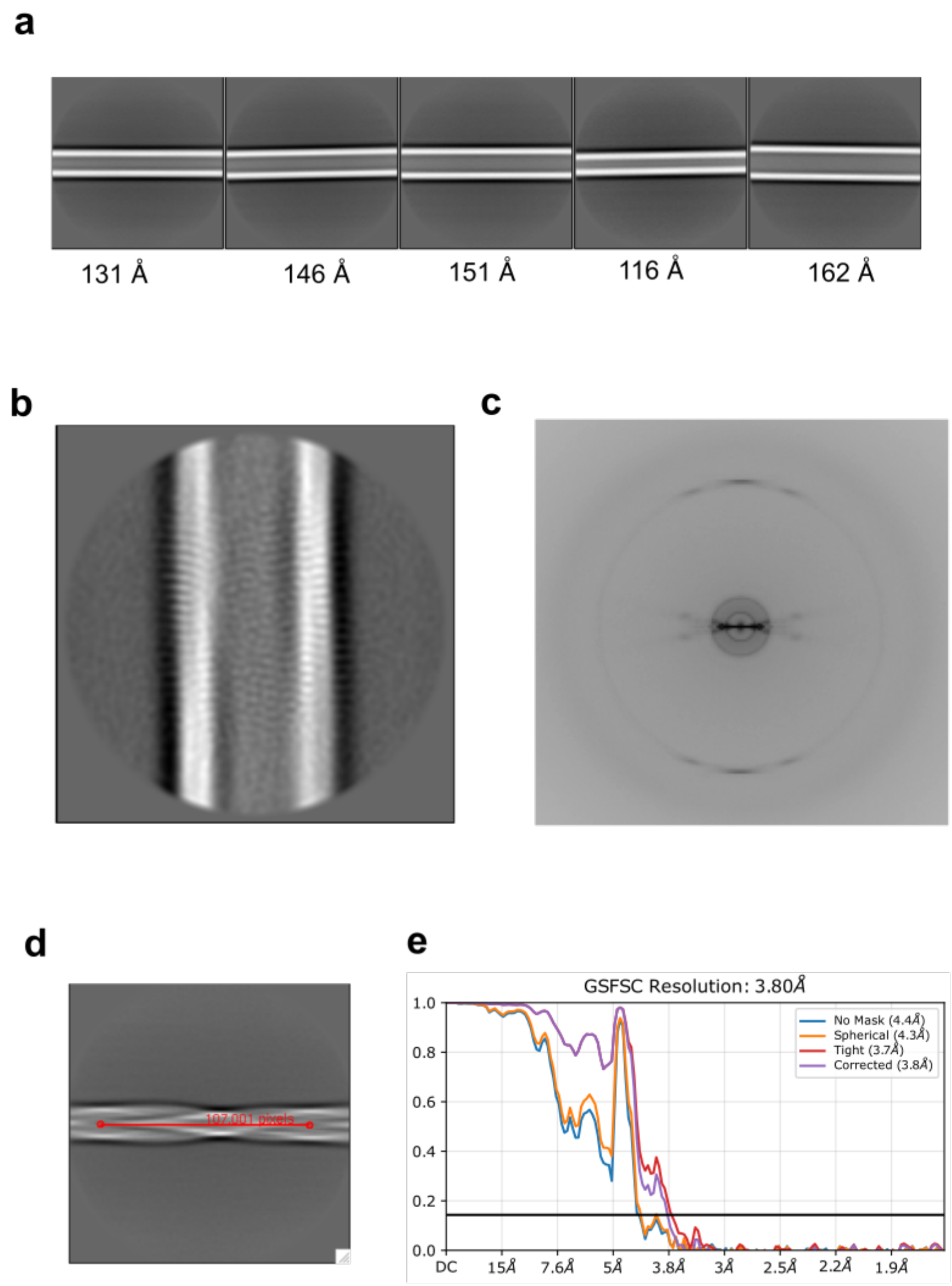

Figure S13. Cryo-EM analysis of assemblies of peptide **4**. (**a**) 2D classification reveals the presence of highly polymorphic tubes from self-assembly at ambient temperature. (**b**) One tube class, extracted using a smaller box size, exhibited minimal repetitive features. (**c**) The corresponding average power spectra. Although all possible symmetries compatible with the power spectra were tested, we could not confidently resolve the tube classes. Subsequent attempts to reconstruct the classes in cryoSPARC yielded even poorer 2D class averages; we attribute this to a lack of distinct structural features needed to guide the refinement algorithm. Nevertheless, the power spectrum displays a prominent layer line at approximately 1/4.85 Å, suggesting a cross-β-like hydrogen bonding arrangement within the tube protomer. (**d**) Representative 2D class of the peptide **4** filament. To estimate the helical twist, we measured the crossover distance from the 2D classes and generated initial models using relion_helix_inimodel2d to test various crossover distances around this estimate, ultimately finding 580 Å to be the most appropriate value. (**e**) Gold-

standard map-to-map Fourier Shell Correlation (GS-FSC) curve (0.143 criterion) for the peptide **4** filament, calculated via the Validation (FSC) job in cryoSPARC.

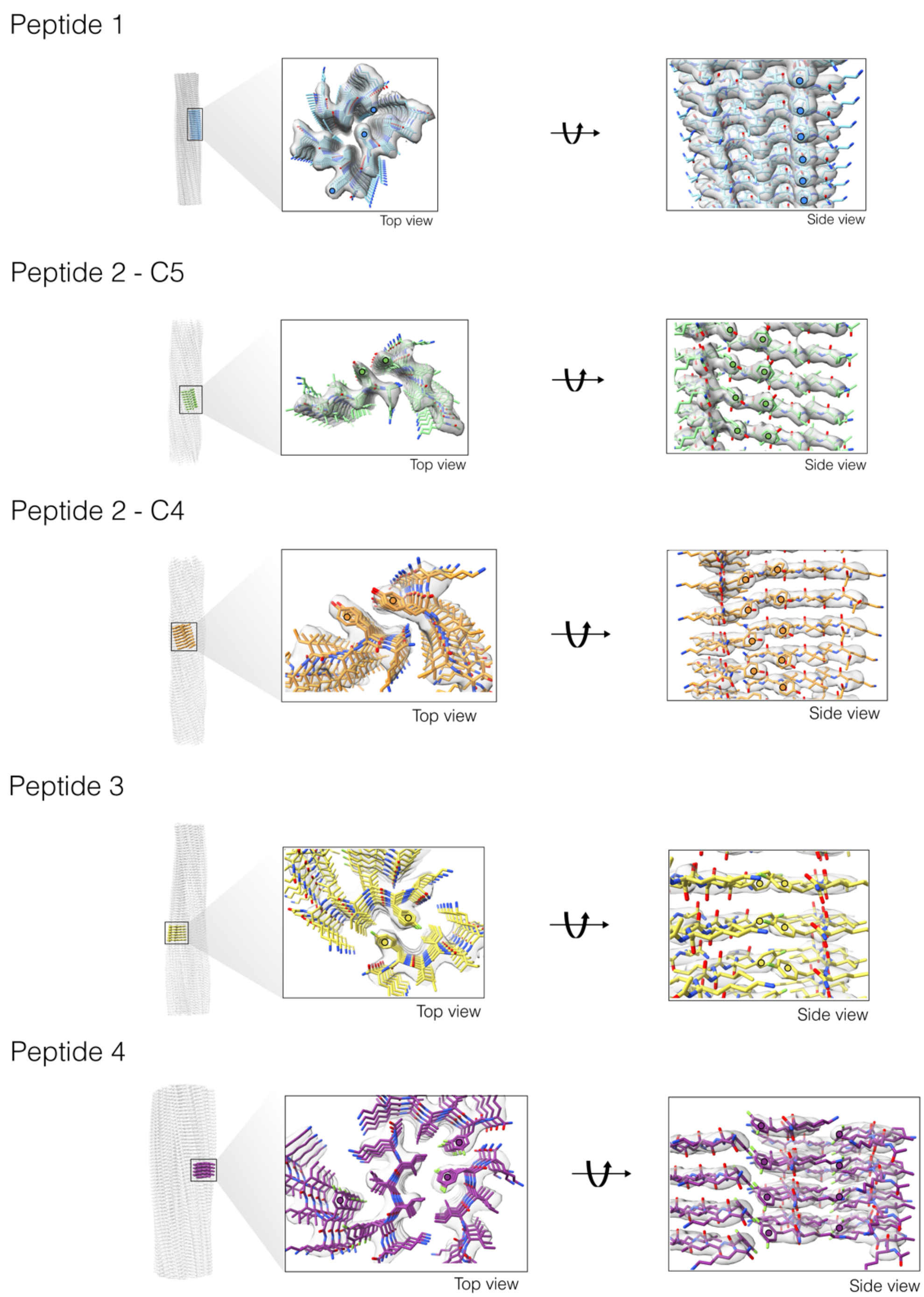

Figure S14. Side-chain density register in the five solved filament structures. Full protofilament reconstruction, with the extracted region boxed (left). Top-down (center) and side views (right) of the density extracted from a single protofilament, shown with the refined model fit. In each structure, the most prominent side-chain density, corresponding to that of the aromatic residue, is marked with a dot colored to match the corresponding model. In the side views, these densities are aligned and in register along the filament axis, as expected for a parallel, in-register strand arrangement.

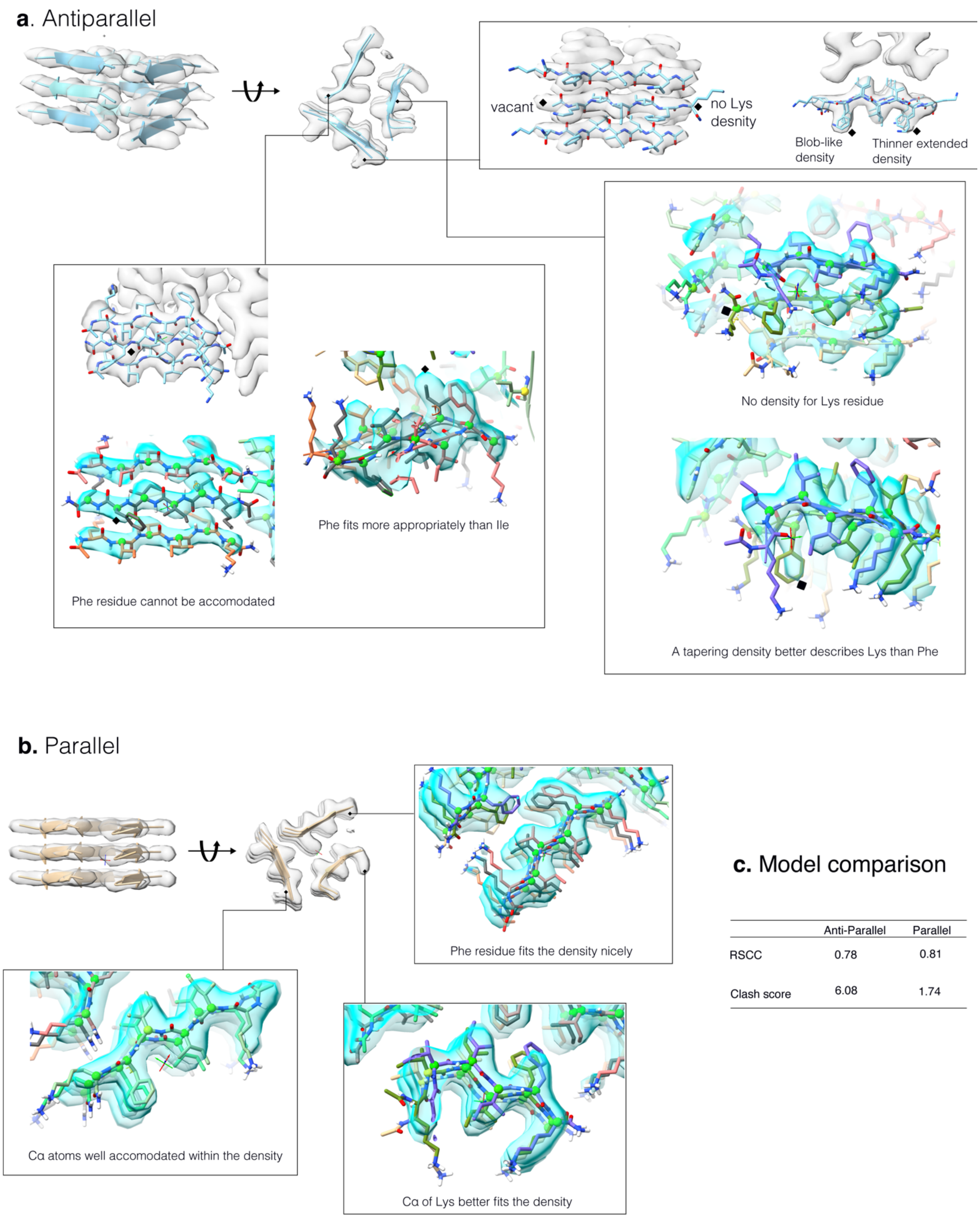

	Anti-Parallel	Parallel
RSCC	0.78	0.81
Clash score	6.08	1.74

Figure S15. Comparison of antiparallel and parallel strand arrangements for peptide **1**. (**a**) Antiparallel and (**b**) parallel models built into three stacked copies of the asymmetric unit, shown

fit to the experimental density. The antiparallel model cannot be fit satisfactorily, as side-chain density is unoccupied, and portions of the backbone are placed in regions lacking supporting density (arrowheads). Neither discrepancy is observed for the parallel model. (**c**) Map–model fit refinement statistics for the two models. Both the real-space correlation coefficient (RSCC) and Clash score values are more favorable for the parallel model.

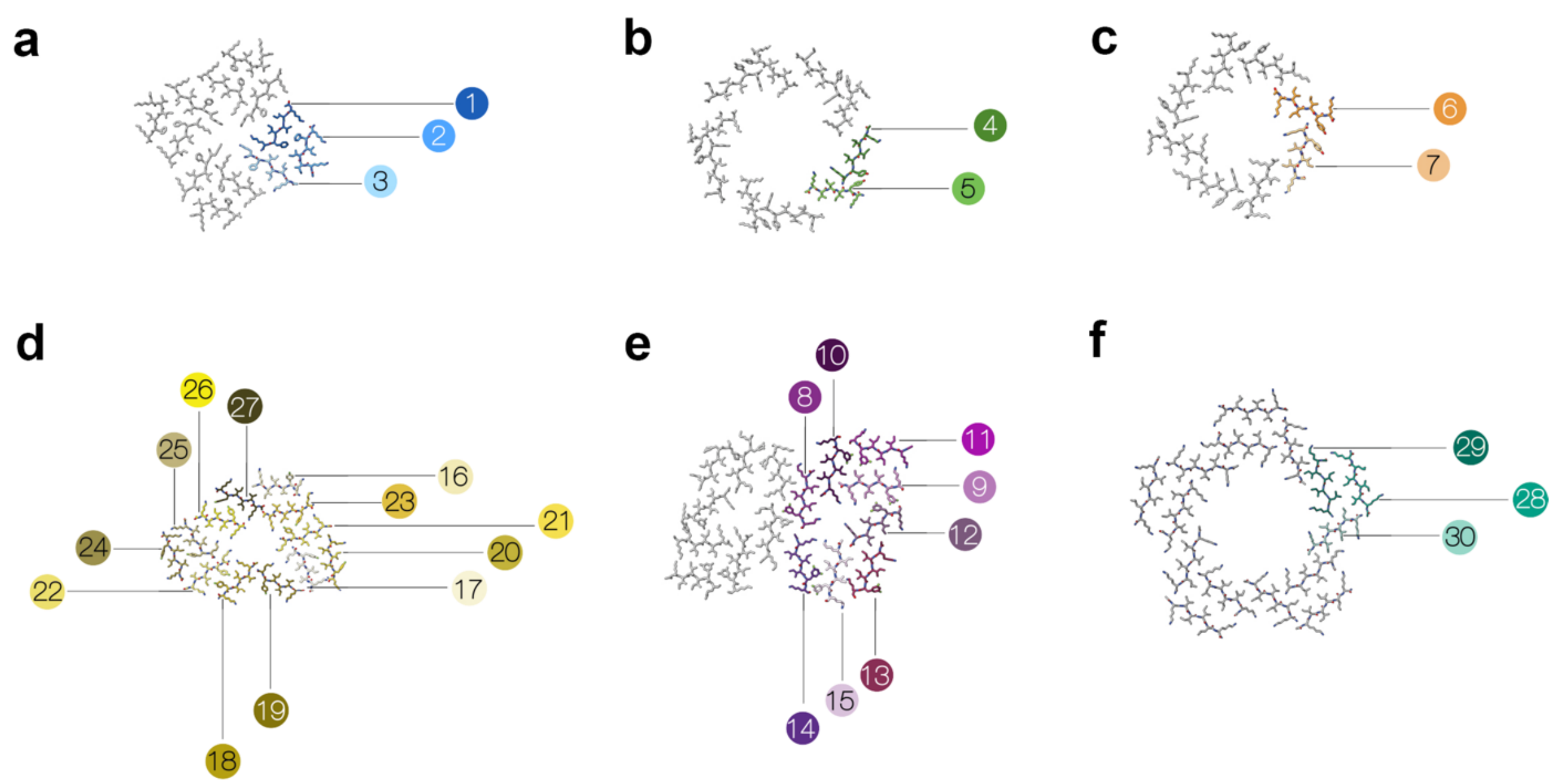

Figure S16. Color-coding of symmetry independent peptides Ac-Lys-Ile-Ile-Ile-**Xaa**-Lys-NH_2 within cross-sectional layers of the respective filaments. RMSD values from pairwise comparisons were employed for construction of the pseudo-phylogenetic tree in Figure 3A. (**a**) Xaa = Phe, (**b-c**) Xaa = Tyr, (**d**) Xaa = 4F-Phe, (**e**) Xaa = 3,5F-Phe, and (**f**) Xaa = Leu. The corresponding PDB IDs are 9D2N (**a**), 9NFN (**b**), 9NGK (**c**), 13MF (**d**), 13FM (**e**), and 9CZ3 (**f**).

Table S1. Cryo-EM data collection and refinement statistics for self-assembled bola-amphiphilic peptide filaments.

Parameter	Peptide **2**-C4	Peptide **1**	Peptide **2**-C5	Peptide **3**	Peptide **4**
Data collection and processing					
Voltage (kV)	200	200	200	300	200
Electron exposure (e-/ Å^2)	57	48.2	57	50	50
Pixel size (Å)	0.84	1.1	0.84	1.07	0.84
Particle images (n)	79,589	122,105	64,499	228,138	7069
Helical symmetry					
Point group	C4	C1	C5	C1	C1
Helical rise (Å)	4.727	1.24	4.924	4.787	2.455
Helical twist (°)	-4.015	89.59	-3.039	-1.831	179.19
Map resolution (Å)					
Reconstruction software	cryoSPARC	cryoSPARC	cryoSPARC	RELION 5.0	RELION 4.0
Model:map FSC (0.5)	4.3	4.4	4.3	4.2	3.9
Map:map FSC (0.143)	3.7	3.5	3.6	3.3	3.8
Refinement and Model validation					
Map sharpening method	DeepEMhancer		Global B-factor	EMReady	EMReady
RSCC	0.79	0.82	0.81	0.72	0.86
Bond lengths rmsd (Å)	0.013	0.013	0.013	0.028	0.021
Bond angles rmsd (°)	1.559	1.701	1.448	2.215	1.561
Clashscore	0.0	1.31	0.0	0.33	0.0
Ramachandran Favored (%)	100	100	100	100	100
Ramachandran Outlier (%)	0	0	0	0	0
MolProbity score	0.50	0.86	0.50	0.62	0.50
Deposition ID					
PDB (model)	9NGK	9D2N	9NFN	13MF	13FM
EMDB (Map)	EMD-49387	EMD-46501	EMD-49371	EMD-77153	EMD-77045

REFERENCES

(1) Kuipers, B. J.; Gruppen, H. Prediction of molar extinction coefficients of proteins and peptides using UV absorption of the constituent amino acids at 214 nm to enable quantitative reverse phase high-performance liquid chromatography-mass spectrometry analysis. *J Agric Food Chem* 2007, *55* (14), 5445-5451. DOI: 10.1021/jf070337l.

(2) Punjani, A.; Rubinstein, J. L.; Fleet, D. J.; Brubaker, M. A. cryoSPARC: algorithms for rapid unsupervised cryo-EM structure determination. *Nat Methods* 2017, *14* (3), 290-296. DOI: 10.1038/nmeth.4169.

(3) Zheng, S. Q.; Palovcak, E.; Armache, J.-P.; Verba, K. A.; Cheng, Y.; Agard, D. A. MotionCor2: anisotropic correction of beam-induced motion for improved cryo-electron microscopy. *Nature Methods* 2017, *14* (4), 331-332. DOI: 10.1038/nmeth.4193.

(4) Sanchez-Garcia, R.; Gomez-Blanco, J.; Cuervo, A.; Carazo, J. M.; Sorzano, C. O. S.; Vargas, J. DeepEMhancer: a deep learning solution for cryo-EM volume post-processing. *Communications Biology* 2021, *4* (1), 874. DOI: 10.1038/s42003-021-02399-1.

(5) He, J.; Li, T.; Huang, S.-Y. Improvement of cryo-EM maps by simultaneous local and non-local deep learning. *Nature Communications* 2023, *14* (1), 3217. DOI: 10.1038/s41467-023-39031-1.

(6) Schäfer, J.-H.; O'Neill, R. T.; Donnelly, J. P.; Powers, E. T.; Kelly, J. W.; Lander, G. C. Helical Reconstruction of Amyloids in cryoSPARC. *bioRxiv* 2025, 2025.2010.2003.680389. DOI: 10.1101/2025.10.03.680389.

(7) Scheres, S. H. W. RELION: Implementation of a Bayesian approach to cryo-EM structure determination. *Journal of Structural Biology* 2012, *180* (3), 519-530. DOI: https://doi.org/10.1016/j.jsb.2012.09.006.

(8) Scheres, S. H. W. Amyloid structure determination in RELION-3.1. *Acta Crystallogr D Struct Biol* 2020, *76* (Pt 2), 94-101. DOI: 10.1107/S2059798319016577.

(9) Lovestam, S.; Scheres, S. H. W. High-throughput cryo-EM structure determination of amyloids. *Faraday Discuss* 2022, *240* (0), 243-260. DOI: 10.1039/d2fd00034b.

(10) Pettersen, E. F.; Goddard, T. D.; Huang, C. C.; Meng, E. C.; Couch, G. S.; Croll, T. I.; Morris, J. H.; Ferrin, T. E. UCSF ChimeraX: Structure visualization for researchers, educators, and developers. *Protein Science* 2021, *30* (1), 70-82. DOI: https://doi.org/10.1002/pro.3943 (acccessed 2024/05/21).

(11) Croll, T. ISOLDE: a physically realistic environment for model building into low-resolution electron-density maps. *Acta Crystallographica Section D* 2018, *74* (6), 519-530. DOI: doi:10.1107/S2059798318002425.
(12) Liebschner, D.; Afonine, P. V.; Baker, M. L.; Bunkoczi, G.; Chen, V. B.; Croll, T. I.; Hintze, B.; Hung, L.-W.; Jain, S.; McCoy, A. J.; et al. Macromolecular structure determination using X-rays, neutrons and electrons: recent developments in Phenix. *Acta Crystallographica Section D* 2019, *75* (10), 861-877. DOI: doi:10.1107/S2059798319011471.
(13) Davis, I. W.; Leaver-Fay, A.; Chen, V. B.; Block, J. N.; Kapral, G. J.; Wang, X.; Murray, L. W.; Arendall, W. B., III; Snoeyink, J.; Richardson, J. S.; et al. MolProbity: all-atom contacts and structure validation for proteins and nucleic acids. *Nucleic Acids Research* 2007, *35* (suppl_2), W375-W383. DOI: 10.1093/nar/gkm216 (acccessed 7/11/2025).
(14) Eaton, D. A. R. Toytree: A minimalist tree visualization and manipulation library for Python. *Methods in Ecology and Evolution* 2020, *11* (1), 187-191. DOI: https://doi.org/10.1111/2041-210X.13313.
(15) Lawrence, M. C.; Colman, P. M. Shape Complementarity at Protein/Protein Interfaces. *Journal of Molecular Biology* 1993, *234* (4), 946-950. DOI: https://doi.org/10.1006/jmbi.1993.1648.
(16) Sulyok-Eiler, M.; Harmat, V.; Perczel, A. Unravelling the Complexity of Amyloid Peptide Core Interfaces. *J Chem Inf Model* 2024, *64* (22), 8628-8640. DOI: 10.1021/acs.jcim.4c01479.
(17) Tien, M. Z.; Meyer, A. G.; Sydykova, D. K.; Spielman, S. J.; Wilke, C. O. Maximum allowed solvent accessibilites of residues in proteins. *PLoS One* 2013, *8* (11), e80635. DOI: 10.1371/journal.pone.0080635.
(18) Sawaya, M. R.; Hughes, M. P.; Rodriguez, J. A.; Riek, R.; Eisenberg, D. S. The expanding amyloid family: Structure, stability, function, and pathogenesis. *Cell* 2021, *184* (19), 4857-4873. DOI: https://doi.org/10.1016/j.cell.2021.08.013.
